# Portable X-ray fluorescence (pXRF) analysis of heavy metals in contaminated soils: a case study from Varta Insel, Hagen

**DOI:** 10.1007/s10653-026-03538-5

**Published:** 2026-10-27

**Authors:** Julius Kaulingfrecks, David De Vleeschouwer, Anja Michalski, Christine Achten

**Affiliations:** 1 Institute of Geology and Paleontology, University of Münster, Correnstraße 24, 48149 Münster, Germany; 2 Arcadis Germany B.V. & Co. KG, Europaplatz 3, 64293 Darmstadt, Germany

**Keywords:** pXRF, Urban soil, Remediation, On site analysis, Heavy metals

## Abstract

Urban soils contaminated with persistent and toxic heavy metals are found worldwide. Their remediation is essential to prevent harm to humans and the environment. The integration of portable X-ray fluorescence spectroscopy (pXRF) as a field analytical instrument in remediation projects offers the possibility to rapidly assess soils at low costs. In this study, we test the potential of pXRF in contaminated soil assessment at a former battery factory “Varta Insel” in Hagen, Germany. Soil samples were analyzed (1) by pXRF *on site*, (2) by pXRF under laboratory conditions, and (3) by microwave assisted aqua regia digestion with inductively coupled plasma optical emission spectroscopy (ICP-OES). Direct on-site measurements were useful for supporting field decisions and selecting samples for further analysis, but results were strongly influenced by matrix effects, grain size, and moisture content, limiting their reliability. Dried and sieved samples were measured by pXRF under laboratory conditions through low-density polyethylene sample bags. The laboratory pXRF results agreed with the more expensive ICP-OES analysis in 94% of cases when classifying samples above or below German regulatory limits. However, pXRF systematically underestimated lead concentrations and overestimated arsenic and chromium. Hence, we conclude that pXRF is not a stand-alone solution for heavy metal analysis of contaminated soil samples. However, it can effectively guide the selection of samples requiring detailed ICP-OES analysis and contribute to higher-density data collection, ultimately enhancing spatial resolution.

**Supplementary Information:**

The online version contains supplementary material available at https://doi.org/10.1007/s10653-026-03538-5.

## Introduction

Since industrialization, environmental pollutants are ubiquitous around the globe. Soils contaminated with heavy metals such as arsenic (As), cadmium (Cd), chromium (Cr), lead (Pb), mercury (Hg), and nickel (Ni) pose severe risks to ecosystems and human health. Heavy metals persist in soils over long periods and can enter the food chain, where they accumulate and become toxic or even lethal (Ali et al., 2019); (Mitra et al., 2022). Although these elements occur naturally, elevated concentrations are often associated with anthropogenic sources such as fossil fuel combustion, industrial processes, mining, or fertilizers (Ali et al., 2019). Urban- and industrial areas are more often affected by significant heavy metal contamination due to their proximity to anthropogenic sources (Wei & Yang, 2010).

Heavy metal contamination is often heterogeneous, and remediation of affected sites is essential to protect residents and the environment. Rehabilitation strategies include containment, removal, and stabilization, with practical approaches ranging from soil replacement and washing (Dermont et al., 2010) to more innovative methods such as asymmetrical alternating current electrochemistry (Xu et al., 2019) or phytoremediation (Gaggero et al., 2020). However, remediation projects are typically lengthy and expensive, sometimes extending over decades with costs reaching $500 per ton of soil (Liu et al., 2018). Effective remediation therefore requires detailed geospatial information on contaminant concentrations, which can only be achieved through large-scale sampling and analysis campaigns. In this context, the choice of analytical method is critical for success (Kalnicky & Singhvi, 2001).

Conventional laboratory methods use microwave-assisted aqua regia digestion followed by inductively coupled plasma optical emission spectroscopy (ICP-OES) or mass spectrometry (ICP-MS). These techniques provide accurate multi-element data but are time-consuming, costly, and limited in throughput. Moreover, aqua regia digestion is the standardized procedure for analyzing heavy metals but can lead to incomplete extraction of certain metals, because metal silicates are not completely dissolved, raising concerns about quantifying total concentrations (Caporale et al., 2018; Kilbride et al., 2006). Thus, the aqua regia digestion is only a partial extraction depending on the composition of the sample. The accuracy is affected by different matrices and varying silica or refractory oxide content (Gaudino et al., 2007). An aqua regia digestion failed to correctly quantify more than 20 elements, especially potassium, aluminum and elements that are strongly bound in clay mineral components (Krause et al., 1995). However, the residual phase is mostly bound to silicate lattices and therefore considered unimportant to evaluate the mobility and behavior of the residual elements (Niskavaara et al., 1997). Mercury can be challenging to be detected at low concentrations using ICP-OES. Other methods such as cold vapor atomic absorption spectroscopy (CVAAS) or ICP-MS can offer reliable quantification (Ghaedi et al., 2006; Mercader-Trejo et al., 2005). While highly reliable, these laboratory approaches restrict the number of samples that can be analyzed, and consequently the spatial resolution of contamination maps.

Portable X-ray fluorescence (pXRF) spectrometry offers an attractive alternative for rapid, in-field analysis. Since its early applications in the 1980s (Bernick et al., 1995; Patten et al., 1982) and recognition by the U.S. EPA as a standard method for inorganic analytes (U.S. EPA), pXRF technology has steadily improved. Handheld devices are cost-effective, require minimal sample preparation, and can operate under field conditions, enabling high sampling densities and supporting on-site decision-making (Lemière, 2018). pXRF has been applied across diverse fields including mineral exploration, waste management, geochemistry, and archaeology (Frahm & Doonan, 2013; Havukainen et al., 2019).

However, the method has clear limitations compared to laboratory techniques. Its higher detection limits, lower accuracy, and reduced precision restrict its stand-alone applicability (Espinoza-Quiñones et al., 2018). Results can be affected by sample homogeneity, sample volume, humidity, analysis time, instrument stability during analyses, analyte interferences, detector resolution and instrument drift (Ravansari et al., 2020). Inter-instrument variability between manufacturers also complicates standardization (Brand & Brand, 2014). Studies consistently show that proper sample preparation (drying, grinding, sieving, homogenization) improves agreement with laboratory results, while unprepared in-field measurements carry greater uncertainty (Shefsky, 1997; Li et al., 2021). Despite these limitations, pXRF can reduce sampling bias, support dynamic field campaigns, and lower overall costs when used in combination with confirmatory laboratory analyses (Taylor et al., 2004; Carr et al., 2008).

Although the potential of pXRF for contaminated site assessment is increasingly recognized, it remains underutilized in remediation projects. Regulatory frameworks tend to favor well-established laboratory methods, and uncertainties remain regarding the comparability of pXRF to ICP-based techniques under realistic remediation conditions. The objective of this study is to assess the potential of pXRF for assessing heavy metal contamination in soils within a remediation context. Specifically, we tested its performance at the site of a former battery factory in Hagen, focusing on three aspects: (1) the effect of measuring through plastic sample bags, (2) the difference between field and laboratory pXRF analyses, and (3) the comparability of pXRF results with those obtained by ICP-OES and CVAAS.

## Materials and methods

### Study area

**Fig. 1:**
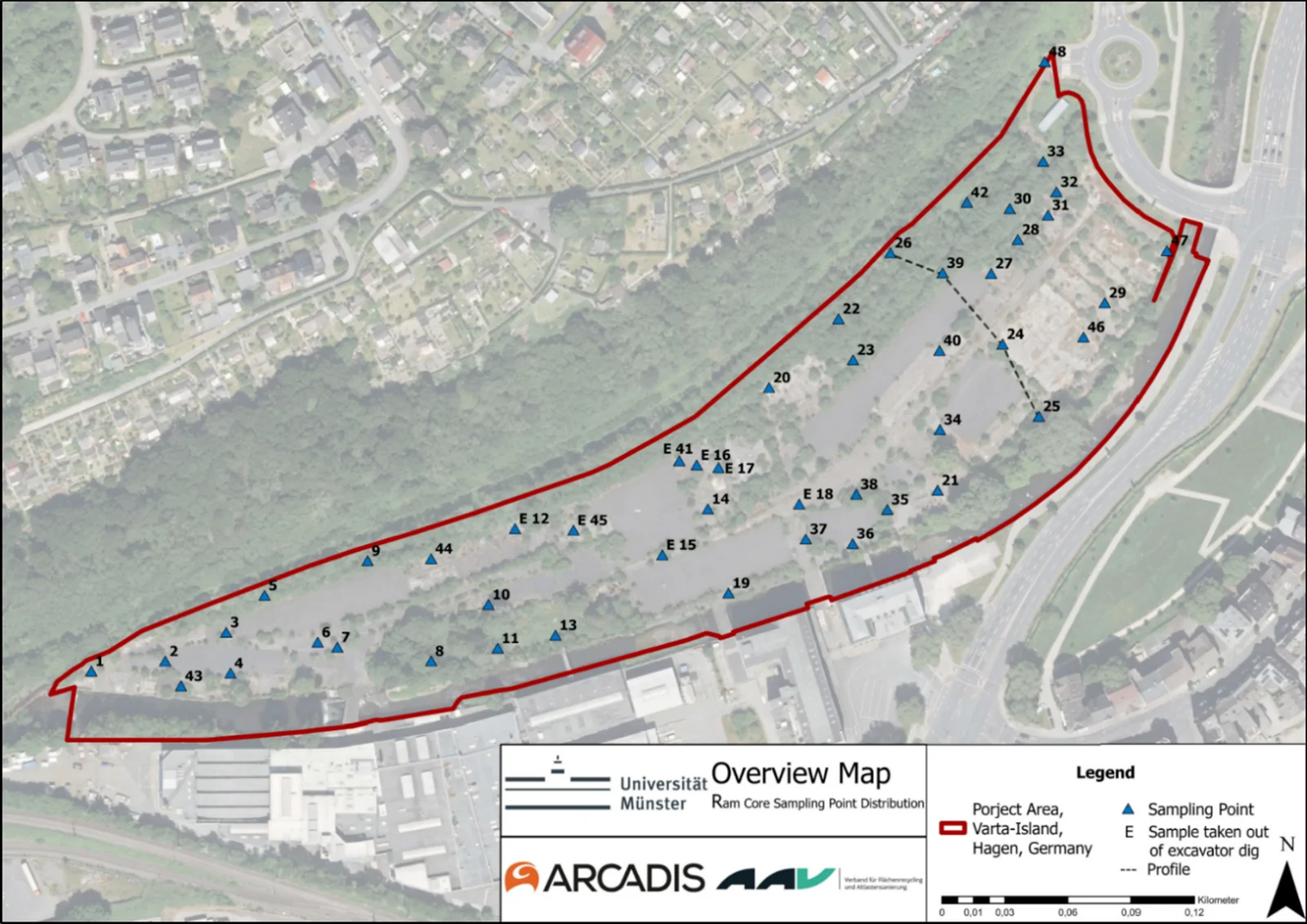
Satellite overview of the study area with distribution of the sampling points and location of the profile section (Fig. 2). Sampling points are spread to represent the entire project area and to hit suspected areas of contamination

Soil samples were taken from the “Varta Insel”, a former battery factory in Hagen, Germany, southwest of the city center (Fig. 1).

The former industrial site has a size of approximately 57,000 m^2^ with nearby access to a highway and railroad. Along its southern boundary, the area is enclosed by the Ennepe River, which flows into the Ruhr River about 5 km to the north. The northern end of the area is marked by an abandoned railroad track. The factory was in operation from 1880 to 2005. In 2011, all buildings were demolished and the area was subsequently sealed with tarmac and concrete. Most of the existing basements and pits were filled with rubble from the demolition. The predominant production process was the manufacture of industrial and automotive batteries, which is why the main soil pollutants include heavy metals. In particular lead, nickel, and cadmium are expected, as they were the most used raw materials. The production of war goods such as submarine batteries in the twentieth century meant that the site was repeatedly bombed during the Second World War. The bombings may have added additional contamination such as oil, heavy metals, or nitroaromatic explosives (Certini et al., 2013).

**Fig. 2:**
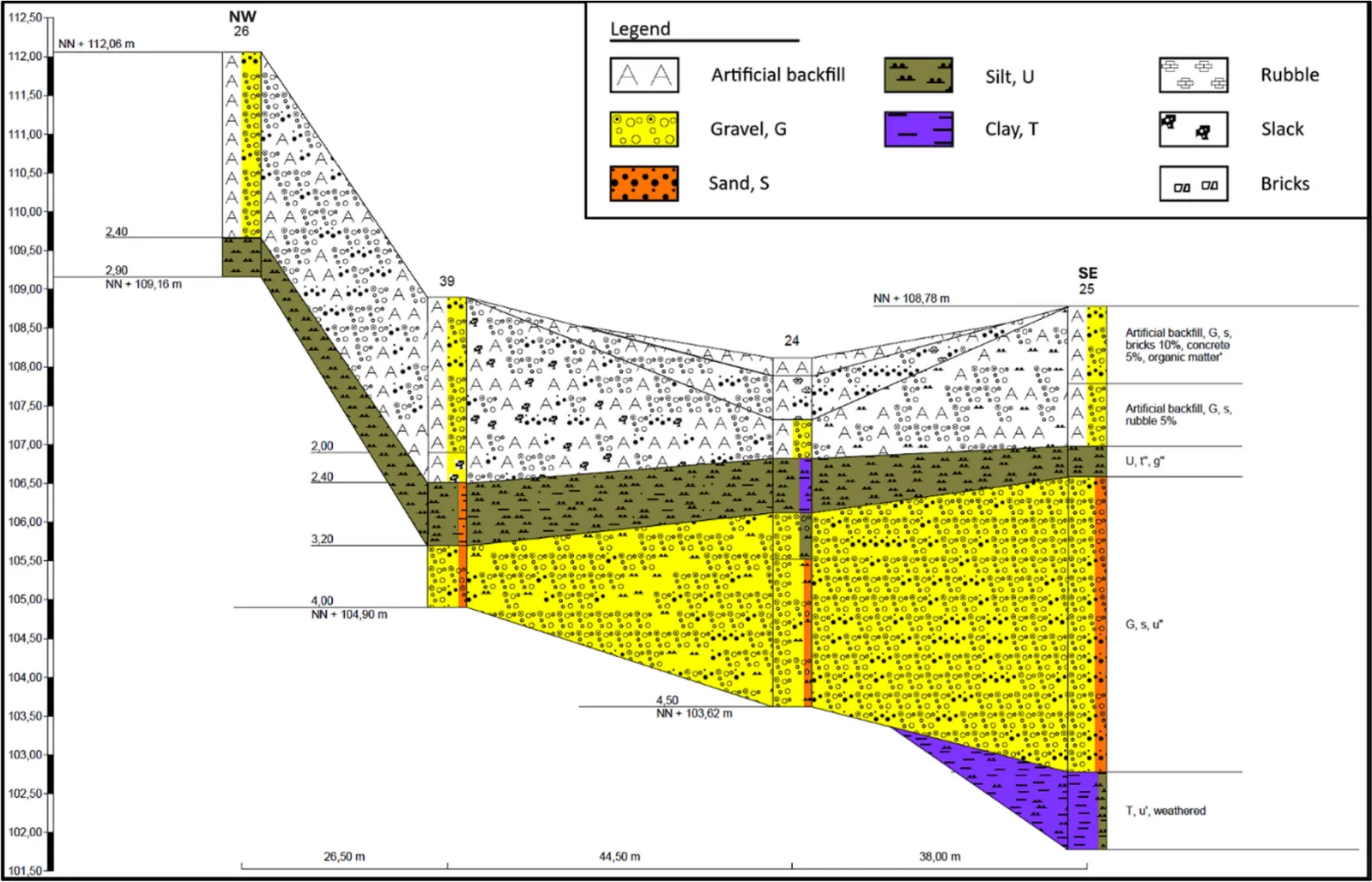
Profile section of the eastern part of the study area as seen in Fig. 1. In the first meters of depth, there is an artificial backfill consisting mostly of gravel and sand with varying amounts of rubble, concrete, broken brick, or concrete. Below the backfill there is natural material in the form of a layer of silt on top of gravel and sand. At the bottom, there is weathered claystone, which was rarely reached as on sampling point 25 in this profile

The top layer of the study area is a surface sealing consisting of tarmac and concrete with a maximum thickness of 1.1 m. Artificial backfill with a maximum thickness of 4.1 m is found below the surface. The backfill is heterogenous and consists of sand, rubble, slag, broken bricks, and occasionally wood, plastic, or metal. The soil is coarse and poorly sorted with grain sizes mostly between medium sand and fine gravel. From previous explorations of the site, it is expected that the heavy metal contamination does not exceed the artificial backfill into the geogenic soil. Under the filling there are quaternary sediments in the form of sandy or silty gravel river deposits with a maximum thickness of 3.5 m that are partially superimposed by flood sediments with varying thicknesses of 0.4–1.3 m. The bedrock consists of Upper Carboniferous claystone (Fig. 2).

### Ram core probing and sampling

A total of 230 soil samples at 48 sample points (Fig. 1) were taken via ram core probing with a diameter of 50 mm up to a depth of 7.4 m and by excavator digs with a maximum depth of 1.2 m. Soil samples were collected out of the 2 m hardened steel drill cores. Samples were taken at least once for every drilled meter, when a different layer occurred before reaching the next meter, or when the soil changed noticeably. The final length of each sampling point was determined by the depth of the groundwater fluctuation zone and the Carboniferous claystone basement. This resulted in an average drilling depth of 4–5 m. The sampling sites were evenly distributed (Fig. 1) to represent the entire area, to fill in gaps of previous explorations, and to hit suspected contamination areas.

Eight ram core probing points were analyzed by pXRF *on site*, with a total of 75 measurements. These measurements were taken immediately on the cut soil material core surface, without any pre-treatment.

All 48 sampling points (ram cores and excavator digs) were sampled for subsequent pXRF analysis under laboratory conditions. Soil samples were therefore collected at one-meter drilling intervals, mixing soil material from the respective one-meter interval. Prior to analyzing, each sample was air-dried for at least 48 h in a laboratory fume cupboard with eightfold air exchange, until there was no more moisture visible. Clumps from drying were broken up with a rubber mallet, after which the soil was sieved to 2 mm grain size.

### Portable X-ray fluorescence spectrometry (pXRF)

Geochemical analysis was performed using a Bruker S1 Titan 800 portable X-ray fluorescence (pXRF) instrument with a rhodium anode, a spot size of 8 mm and a graphene detector window. The measurements were performed in three phases, each lasting 30 s. Phase one involved the use of a Ti–Al filter with a voltage set at 30 kV and an electrical current of 12.6 μA. Phase two featured a Cu-Ti–Al filter, with the voltage adjusted to 50 kV and an electrical current of 15.7 μA. The final phase was performed without a filter, employing a voltage of 15 kV and an electrical current of 9.6 μA. Backscattered fluorescent X-rays were detected over a range spanning 0–41 keV. Deconvolution and calibration were performed according to the “GeoExploration—Oxides” Bruker procedure. Before using the instruments, calibration was tested with the manufacturer’s calibration standard. The limits of detection as reported by the manufacturer are listed in the Supplemental Material (see SM.3).

### Experimental setup for quantifying the effect of the sample bag

Measuring samples through a polyethylene layer can affect X-ray fluorescence readings by dampening, amplifying, or scattering the signal. Although X-rays generally pass unaffected through light elements with atomic numbers below 11 (Kikongi et al., 2017), previous studies gave conflicting recommendations: Santana et al. 2018) found no significant effects using 0.01 mm polyethylene bags, while Lemière (2018) advised avoiding any barrier to prevent bias. This study compares low-density polyethylene (LDPE) bags (0.05 mm thick) with standard XRF thin foil (polyethylene microfilm, 0.0015 mm). The samples were covered with a thin foil, which is highly transparent to X-rays while preventing contamination of the measurement window and retaining the sample material during analysis. The LDPE bags are 33 times thicker than the foil and five times thicker than those used by Santana et al. 2018). To test potential bias, an experiment was conducted using commercially available LDPE bags (80 × 120 mm, 0.05 mm thick). Two reference materials (SARM46, JMs-2) and a calibration standard (CS-M2) were analyzed, each measured ten times, repositioning the sample between measurements, with and without the LDPE bag. CS-M2 is the manufacturer’s powdered calibration standard without a complete set of published values. SARM46 (river sediment, MINTEK, South Africa) and JMs-2 (deep-sea sediment mixture, Geological Survey of Japan) were provided by the Institute of Mineralogy, University of Münster, with analytical data taken from the GeoReM database (Jochum et al., 2005). Reference powders were firmly packed in pXRF sample cups with a 1.5 µm polyethylene microfilm.

### Inductively coupled plasma optical emission spectroscopy (ICP-OES)

Of the 230 samples a subset of 113 samples were additionally examined using ICP-OES as described by Tighe et al. (2004). The analysis was performed with the “Thermo Scientific iCAP 6500” by the SEWA Laborbetriebsgesellschaft mbH in Essen, Germany. The standard parameters for heavy metals in soils As, Pb, Cd, Cr, Cu, Ni, Sb, and Zn were analyzed. The samples were dried, ground and subjected to microwave assisted aqua regia digestion as described in the European standard DIN EN 13657:2003-01 “Characterization of waste—Digestion for subsequent determination of aqua regia soluble portion of elements in waste” (DIN EN, 2003). After dissolution, the samples are vaporized in an argon plasma and the resulting characteristic electromagnetic radiation is detected by an optical emission spectrometer (DIN EN ISO, 2009; Olesik, 1991).

### Cold vapor atomic absorption spectroscopy (CVAAS)

In addition to the previous method, the mercury concentration of the soil samples was determined by cold vapor atomic absorption spectrometry (CVAAS), which is a very selective and sensitive method for mercury determination in solid or liquid samples (Ghaedi et al., 2006). The “PerkinElmer FIMS-400” spectrometer was used for this purpose. Mercury analysis is challenging due to its high vapor pressure and matrix effects, so a cold vapor analysis method such as CVAAS is used to prevent analyte loss by not applying additional thermal energy. The samples were dried, ground and subjected to microwave assisted aqua regia digestion, reduced with a reducing agent and vaporized using pure argon (99.998%) as carrier gas (U.S. EPA, 1974). The vapor is excited by an UV-light source. The absorption of light by free mercury ions is then detected by a secondary electron multiplier. The application of CVAAS provides good sensitivity and a limit of detection (LOD) as low as 13.2 ng/g in soil samples (Mercader-Trejo et al., 2005). The Hg samples were taken from the same aqua regia solutions that were prepared for ICP-OES.

### 3D modeling

A three-dimensional model was constructed using Earth Volumetric Studio (EVS, CTech Development Company, USA) to interpolate **lead (Pb) concentration measurement obtained at multiple depths from several drill cores**. The EVS software is a 3D modelling tool used to analyze and visualize subsurface data. The terrain surface was modeled using elevation points from every sampling point and an additional 1,139 measured elevation points. A stratigraphic model consisting of four layers (artificial backfill, silt, gravel and claystone basement) was constructed based on the geological strata identified in the field and the digital terrain surface. Subsurface concentration volumes were interpolated from depth-resolved Pb measurements using a three-dimensional spatial framework. Two **geometric interpolations were** conducted, one for pXRF measurements (227 measurements from 59 drill cores) and one for ICP-OES measurements (224 measurements from 129 drill cores, including historical data). We emphasize that this interpolation does not explicitly simulate physical or chemical processes such as deposition mechanisms, contaminant transport, or attenuation.

Spatial regionalization of Pb concentration data was performed using **ordinary kriging**, as implemented in EVS, assuming a locally constant but unknown mean and spatial autocorrelation defined by an empirical variogram. The model type is spheric. The variogram parameters of the geologic model were a range of 244 and a sill of 2,37, and of the lead concentration had a range of 277–279 and a sill from 0.37 to 0.43 with a nugget of zero. Concentration interpolation was unconstrained by geological boundaries or other geospatial information.

## Results and discussion

### Effect of measurements through plastic bags

**Fig. 3:**
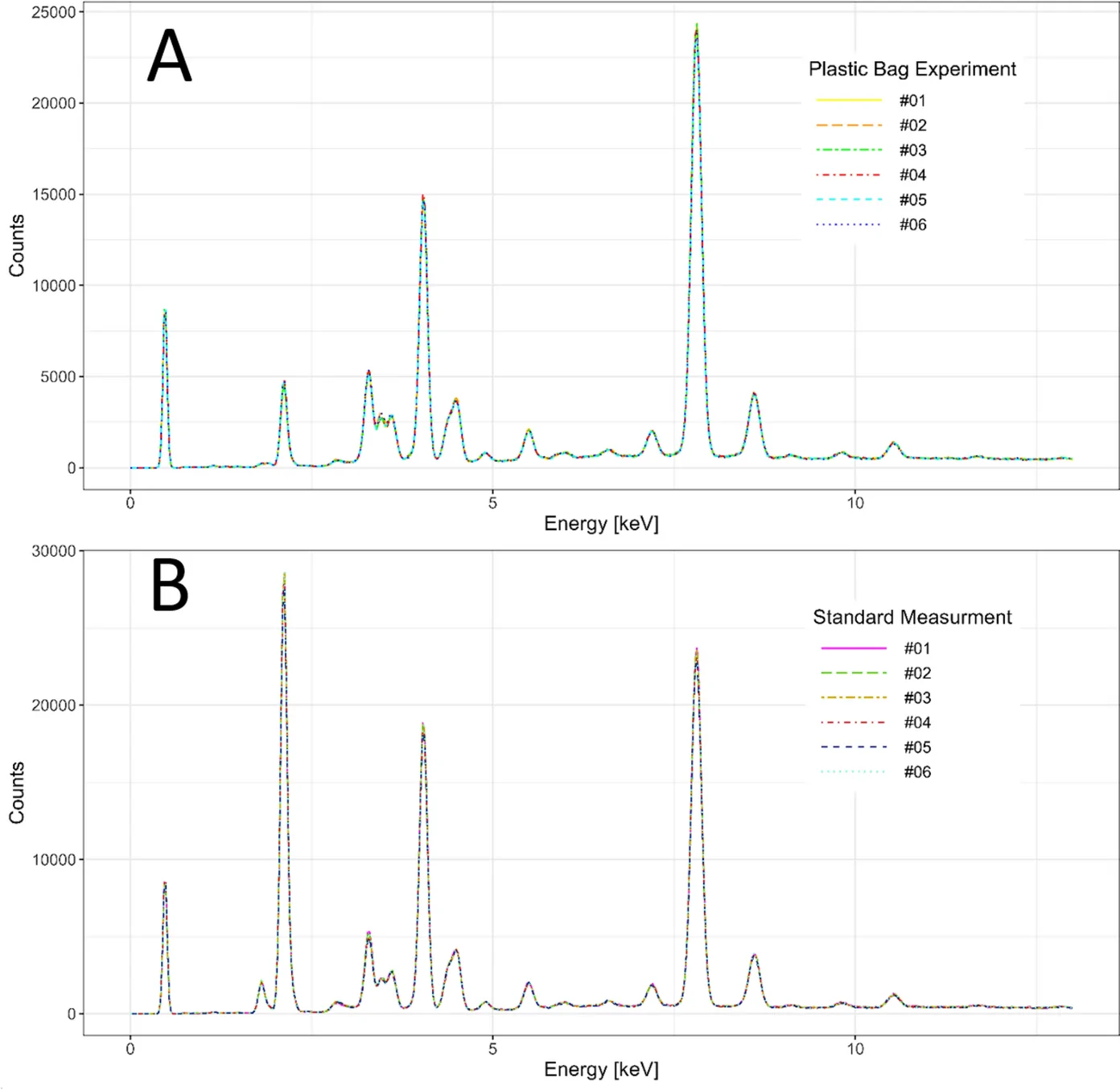
Portable X-ray fluorescence spectrometer spectra (counts vs. energy in keV) of the first six measurements of the manufacturers CS-M2 calibration standard, measured through the low-density polyethylene plastic bag (**A**) and through thin film (**B**). The six spectra show minimal differences, proving that the method is consistent and not affected by chaotic errors

**Fig. 4:**
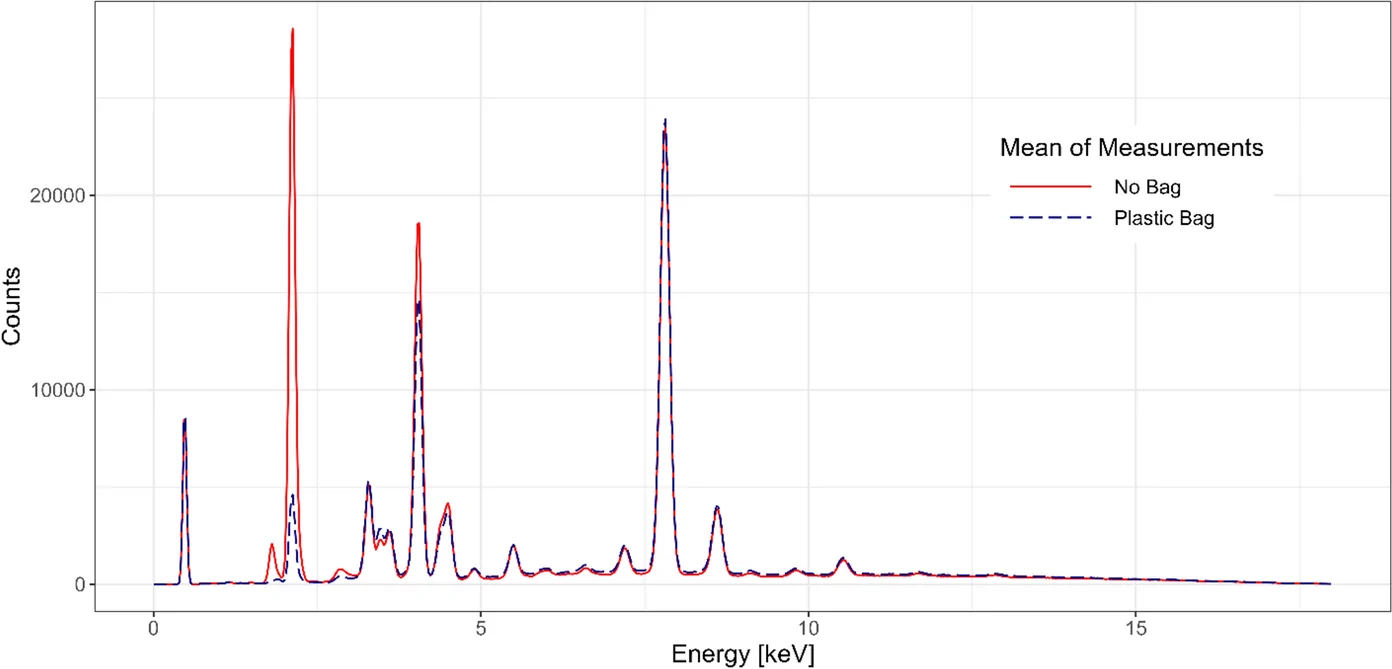
Averaged portable X-ray fluorescence spectrometer spectra (counts vs. energy in keV) of calibration standard measurements from the plastic bag experiment with and without the plastic sampling bag. The spectra show the manufacturers CS-M2 calibration standard, measured through the low-density polyethylene plastic bag (blue, dashed) and through thin film (red)

The pXRF performance can be influenced by any layer of plastic, thin film polymer, or even air between instrument and sample. Measuring the sample through a plastic sampling bag is a practical option to quickly screen a sample in a measurable state with dense packed sediment and little air between the sample and the instrument while minimizing the risk of exposure to and dispersal of hazardous material during handling. A plastic film will attenuate or scatter fluorescence signals to some extent (Parsons et al., 2013). The resulting spectra show no substantial difference between repeated measurements made in the same condition (Figs. 3 and 4). Subsequently, there is little instrumental drift or chaotic error. The most strongly affected elements were the lightest elements and their corresponding molecules MgO, Al_2_O_3_, SiO_2_, P, S, Cl, and K_2_O for which between 50 and 90% of the concentration was lost due to the effect of the LDPE layer. The exact values can be found in the supplemental material. The elements heavier than Potassium (Ca, Ti, Cr, Mn, Fe, Ni, Cu, Zn, As, Rb, Sr, Y, Zr, Cd, Sb, Ba, Hf, Pb) deviate less than 30% from the original value. The signal of some elements, such as Mn, Sr, Y, and Ba, is amplified by the sample bag. For the purpose of heavy metal analysis, the parameters As, Cd, Cr, Cu, Ni, Sb, and Pb are highlighted. Since the individual measurements do not differ much, an average of the ten measurements is calculated for each element and each standard (Fig. 4). There is a significant difference between the averaged spectra obtained in the presence and in the absence of the plastic bag. A much smaller peak at 2.4 keV is observed with the plastic bag, explaining the 90% lower SiO₂ concentration. For each element, a compensation factor was calculated from the difference between the means of the results with and without a sampling bag, taking into account the LOD and how the published values fall within the calibration (Supplemental Material, SM.5). A correction factor for V, Ga, Se, Nb, Mo, Ag, Sn, La, Ce, Ta, W, Pt, Au, Hg, Tl, Bi, Th, and U could not be determined, because the used reference materials do not contain the element, or the element concentration was below or at the devices LOD. In future studies or pXRF applications when measuring through LDPE, a reference material with all heavy metals in the relevant concentration ranges regarding regulatory levels and instrument calibration should be used. The compensation factor corrects only for the effect of the plastic bag. It does not account for the inherent deviation between the thin-film (“no-bag”) pXRF measurements and the published reference materials (GeoReM database (Jochum et al., 2005)). This deviation was deliberately left uncorrected, since including it would risk overcompensating and introducing additional bias into the bag-affected pXRF results. For analyses focusing on the lightest elements, measurements should not be conducted through plastic bags. Otherwise, the measurements with pXRF as described in this study (laboratory conditions, dried samples, measured through the sampling bag with the suggested correction factor applied) provide reliable and accurate results comparable to conventional pXRF results (measured through polyethylene microfilm 1.5 μm thick)). Repeated measurements of the same sample showed negligible benefit (Fig. 3).

### Analyses in the field and in the laboratory

**Fig. 5:**
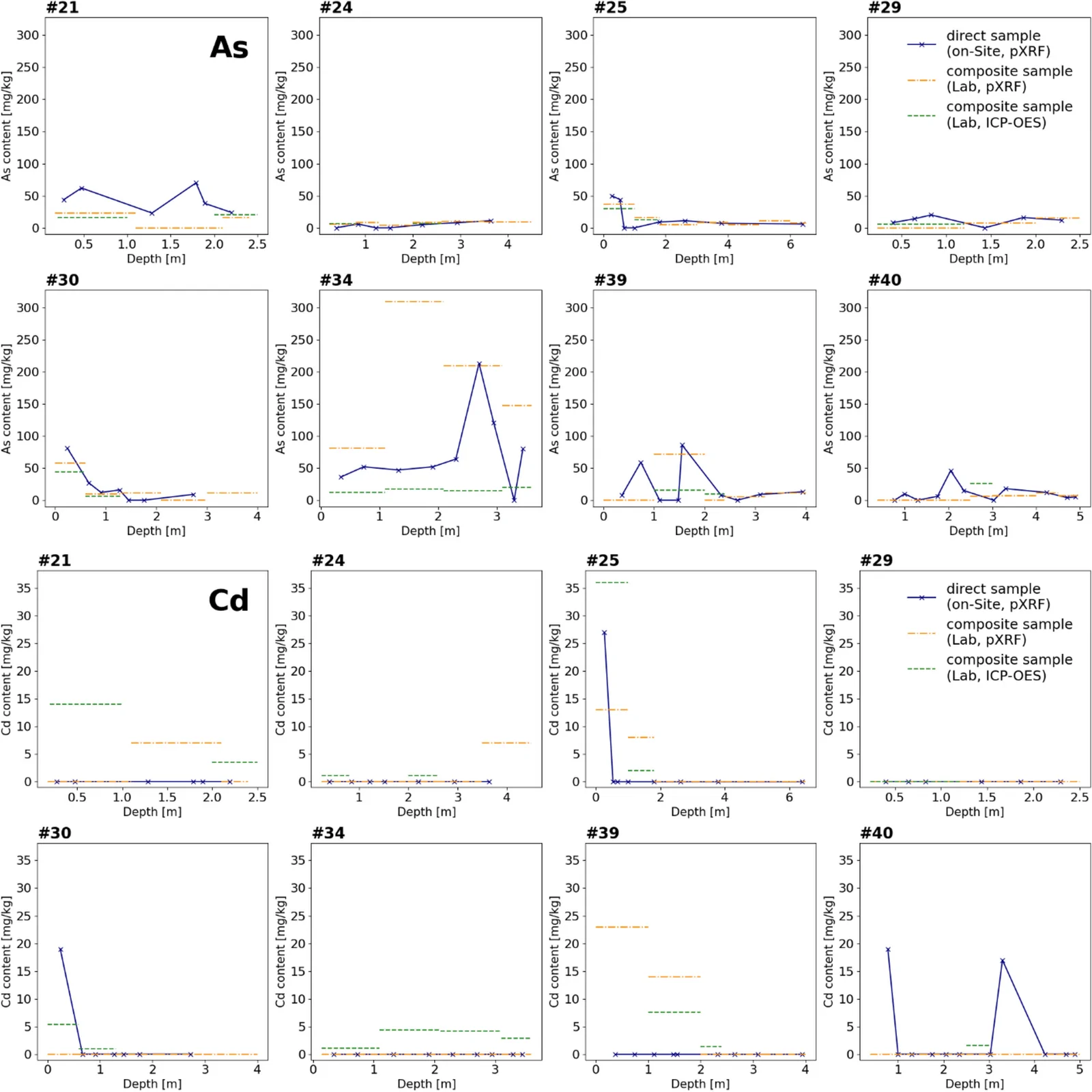
The arsenic (As) and cadmium (Cd) concentrations (mg/kg) in relation to depth (m). All eight sampling points with available on site data are displayed (21, 24, 25, 29, 30, 34, 39, and 40). A comparison of the As on site (pXRF) and laboratory (pXRF, ICP-OES) data shows good consistency between the methods. The largest disagreement is at #34, which could be caused by a sampling or handling error or the very heterogenous composition of the samples. The limit of detection (LOD) of the pXRF for As is 10 mg/kg. The limit of detection (LOD) of the pXRF for Cd is 30 mg/kg, which is higher than all recorded concentrations with the exception of one ICP-OES measurement at #25

**Fig. 6:**
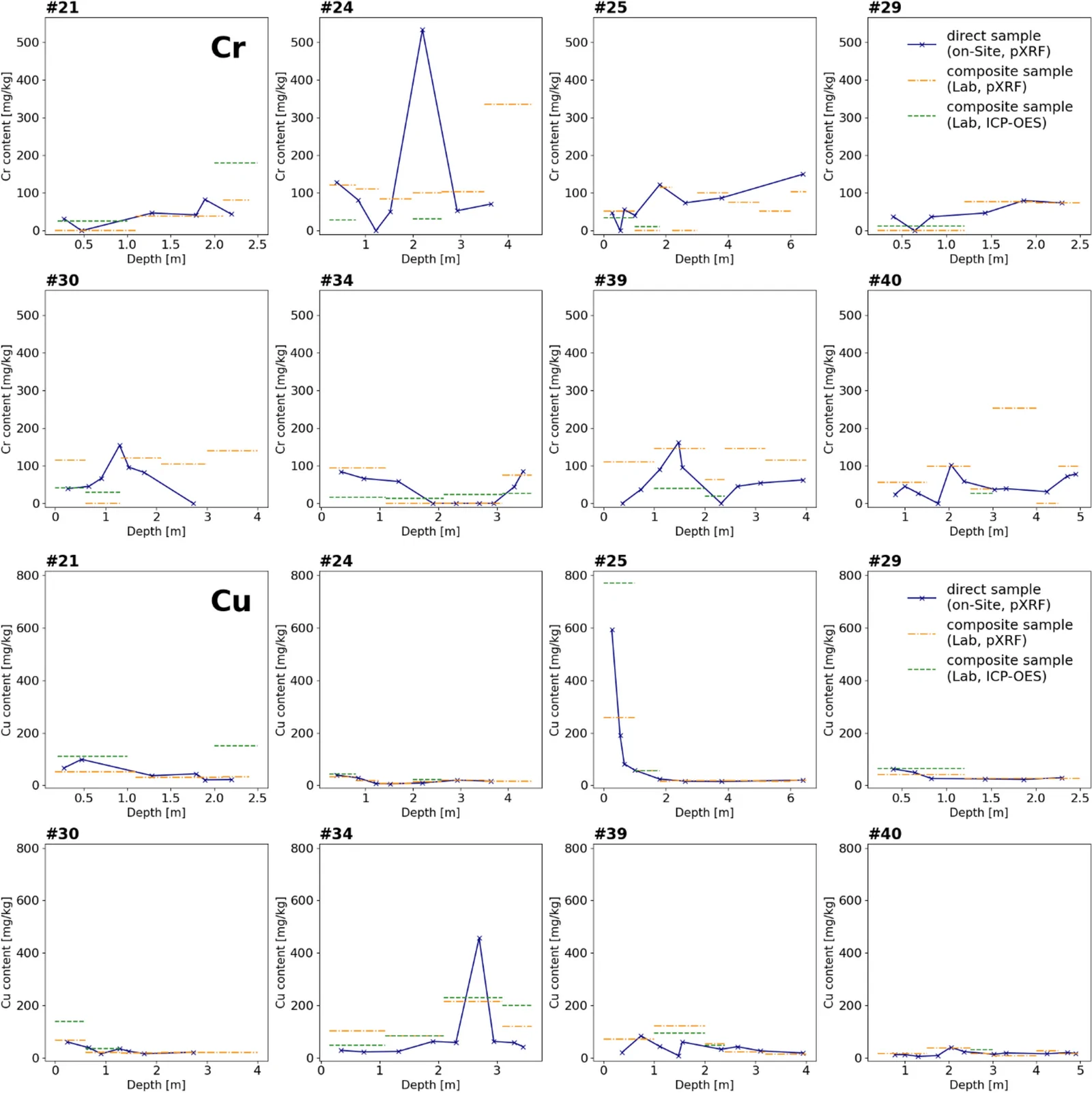
The chromium (Cr) and copper (Cu) concentrations (mg/kg) in relation to depth (m). All eight sampling points with available on site data are displayed (21, 24, 25, 29, 30, 34, 39, and 40). The limit of detection (LOD) of the pXRF for Cr is 140 mg/kg. Most values plot close to that limit. At #24, there are large differences which could be caused by a Cr hotspot only measured on site. Fluctuations under the LOD could be caused by instrumental shift. The LOD of the pXRF for Cu is 30 mg/kg. The ICP-OES, on site-, and laboratory pXRF values fit mostly well

**Fig. 7:**
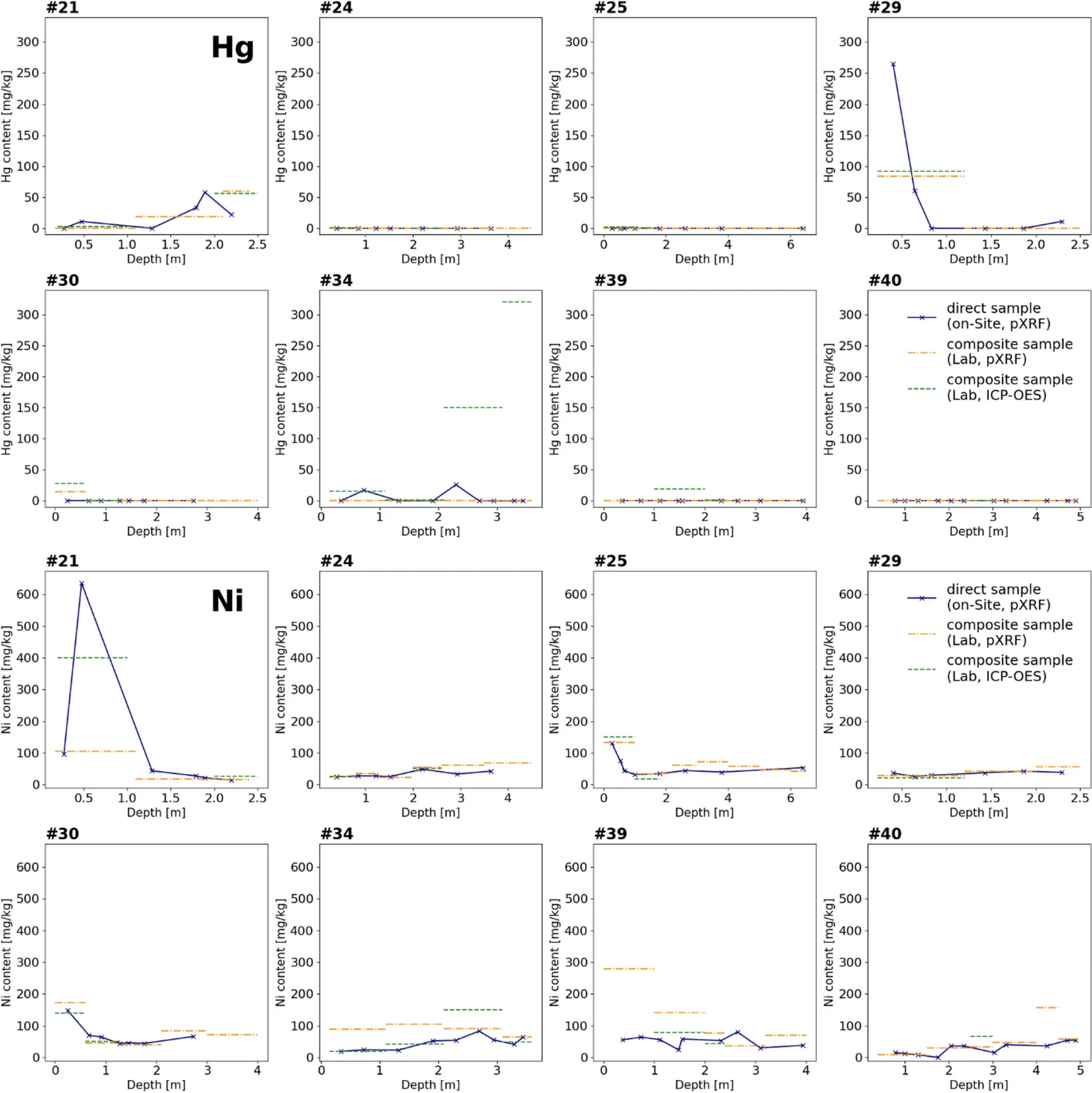
The mercury (Hg) and nickel (Ni) concentrations (mg/kg) in relation to depth (m). All eight sampling points with available on site data are displayed (21, 24, 25, 29, 30, 34, 39, and 40). The limit of detection (LOD) of the pXRF for Hg is 30 mg/kg. The Hg concentrations of the three methods agree well with the exception of two peaks at 34 that were measured only by ICP-OES. The Ni data overall matches well, with a few more deviations. At 21 there is a large difference between ICP-OES and laboratory pXRF, that can be explained by an incomplete homogenization of the pXRF sample since the concentrations fluctuate a lot as seen in the on site pXRF results. The LOD of the pXRF for Ni is 20 mg/kg

**Fig. 8:**
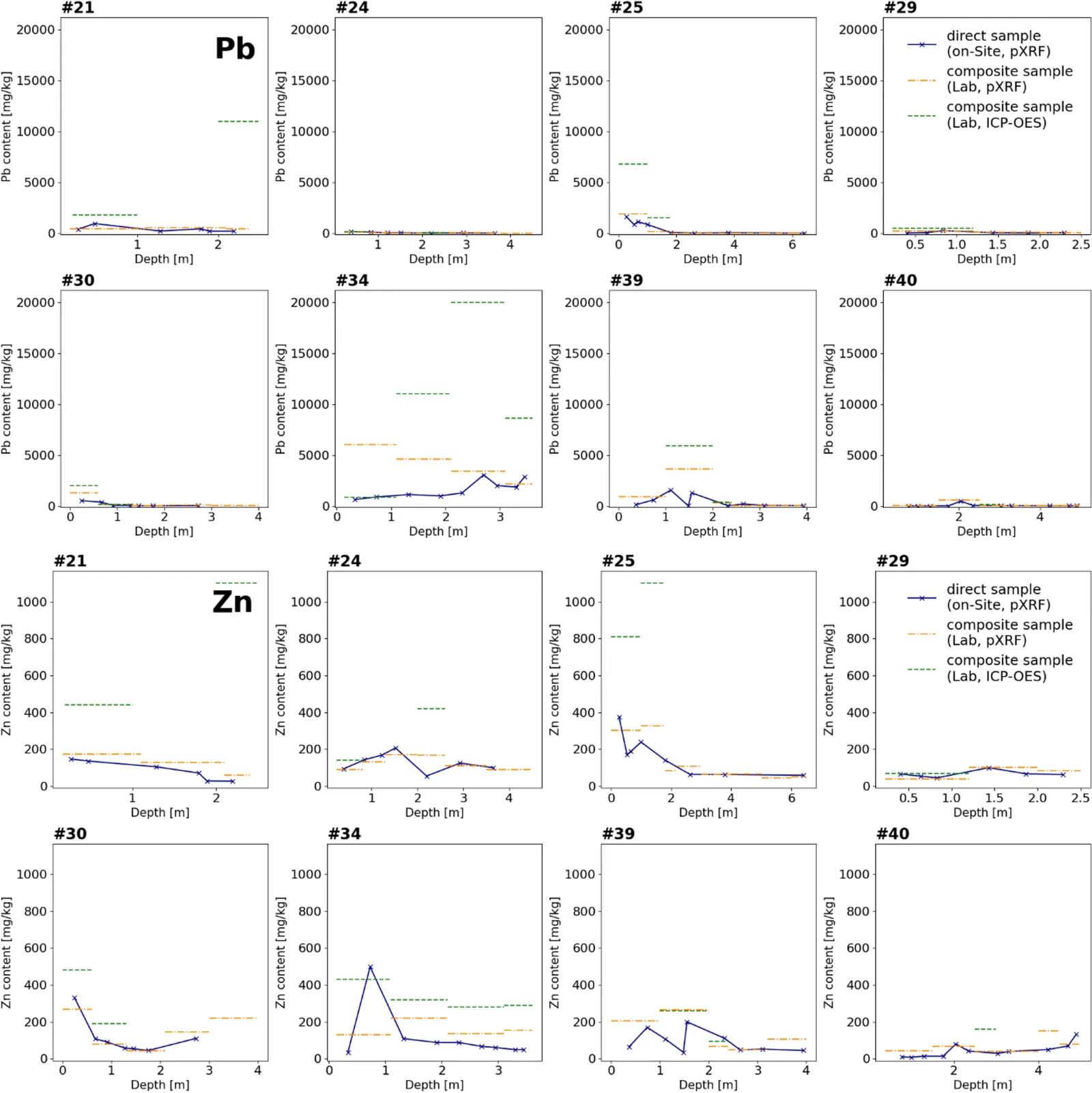
The lead (Pb) and zinc (Zn) concentrations (mg/kg) in relation to depth (m). All eight sampling points with available on site data are displayed (21, 24, 25, 29, 30, 34, 39, and 40). The limit of detection (LOD) of the pXRF for Pb is 20 mg/kg. The pXRF recorded Pb concentrations fit relatively well but are generally lower than the ICP-OES Pb concentrations from a systematic underestimation of Pb with pXRF in this setting. For Zn, the figure also shows similar sets of pXRF results and mostly larger ICP-OES concentrations. This effect is probably caused by heterogenous samples where higher Zn quantities were only revealed by the complete digestion for ICP-OES. The LOD of the pXRF for Zn is 10 mg/kg

Of the total 48 sampling points, 8 were analyzed *on site* with a total of 75 measurements taken directly on the drill core at varying depths. These points were also sampled, as described above, as 43 mixed samples in LDPE bags. All mixed samples were analyzed by pXRF under laboratory conditions, out of which 16 of 43 were also analyzed by ICP-OES (Figs. 5, 6, 7 and 8). The 16 samples were selected based on observable physical abnormalities. These mixed samples or composite samples represent a range of depth from which the sample was collected. The samples that were analyzed in the lab by pXRF and ICP-OES are subsamples from the same original mixed samples. The *on site* measurements showed element concentration at that particular depth. At certain depths no on-field measurements were taken because the water-saturated area was reached. High water content can significantly alter the resulting concentrations (Parsons et al., 2013). Soil humidity can generally be disregarded with an overall minor error when fluorescence energies are higher than 5 keV and the moisture content is below 20% (Kalnicky & Singhvi, 2001). However, sediment or soil samples with greater water content than 20 wt.% should be dried. A signal loss of 37% for arsenic was recorded by Parsons et al. (2013) with samples containing 20 wt.% moisture relative to dried samples. The effect of moisture is different for each element, with elements that have low-energy K-lines more strongly affected than elements with higher-energy K-lines. While the direct samples were not dried before being measured to simulate quick on site screenings, the samples that were measured under laboratory conditions were dried in their preparation.

The highest concentrations of a single element measured by pXRF were found for lead with around 6,000 mg/kg in the mixed sample and around 3,000 mg/kg *on site* at sampling point 34 for which the ICP-OES recorded 20,000 mg/kg at 2.1–3.1 m. The concentrations of the other heavy metals at the eight sampling points were much lower, with Cd staying below 40 mg/kg. The concentrations of As, Hg, and Sb were all under 500 mg/kg and those of Cr, Cu, and Ni as well, each with the exception of a single sample. The median and average concentration of the non-lead contaminants was below 50 mg/kg. The As results showed good agreement between the field analysis and both the pXRF- and ICP-OES lab results, except for an isolated outlier at point 34 and slightly elevated on-site measurements at point 21 (Fig. 5). The same is true for Hg with a good agreement of concentrations except for two peaks at sampling point 34, which were only recorded by CVAAS (Fig. 7). The observed Cd concentrations fit well amid the compared methods even when all recorded concentrations were below the pXRF LOD of 30 mg/kg (Fig. 5). The Cr values were mostly below or at the LOD of 140 mg/kg, some spikes were only reflected in on-field or laboratory results, such as at points 24 and 40 (Fig. 6). The high peaks that were only recorded in the *on site* data, such as Cr at point 24, could be explained by a local hotspot in the heterogenous soil. The recorded Cu concentrations were in notably good agreement (Fig. 6). Similar to Cu, the three sets of Ni concentrations were matching well to each other except for a few individual differences. At sampling point 21 there is a large difference between ICP-OES and lab pXRF in Ni concentration, which could be explained by an incomplete homogenization of the pXRF sample since the local concentrations fluctuate a lot as seen in the *on site* pXRF results (Fig. 7). Comparing the on-site and laboratory pXRF Pb results revealed very similar concentrations, although some laboratory concentrations were higher at points 34 and 39. The ICP-OES results revealed generally higher Pb concentrations than pXRF, with some much larger concentrations at points 21, 25, 34, and 39 (Fig. 8). This could be an effect of a systematic underestimation of Pb with pXRF. The Zn concentrations in Fig. 8 show similar sets of pXRF results with often larger Zn concentrations from ICP-OES. Heterogenous samples with higher Zn contents that were only revealed by the aqua regia digestion could explain this effect. The measured Sb concentrations were almost all below or close to the LOD of 100 mg/kg with only a few samples above at points 25 and 34. The laboratory pXRF results were generally higher than the *on site* results. There were no ICP-OES Sb measurements from the eight sampling points with available *on site* analysis (SM.4).

When comparing the results from the *on site* analysis and the pXRF analysis at laboratory conditions, it is apparent that the two datasets would lead to the same conclusion in most cases. The larger disagreements can be explained by heterogeneity of the soil and sample. The *on site* samples were only mixed and disturbed by the drilling process. The precise distribution and origin of the anthropogenic soil within the project area is unclear. The occasional high heavy metal concentration could have been caused by small patches of soil material with larger concentrations. At sampling point 34, multiple inconsistencies between the three datasets may have arisen from a combination of errors such as sample mix-ups, measurement errors, matrix effects, or sample bag effects. It is important to note that up to 2.1 m depth sampling point 34 contained approximately 60% slag, from 2.1 to 3.6 m it contained almost purely slag. Most likely a heterogenous slag filled pit was sampled with diffuse heavy metal contents, which could explain the uncertain results at point 34.

Within the *on site* data, there are individual measurements with high and low concentrations that are close to each other, with the concentration of the mixed sample falling in between (Figs. 6 and 7, i.e. Cr #24, Cu #34, Ni #21). In such cases, the composite sample likely provides a better overall indication of the area, albeit with reduced vertical resolution. The higher data density of single data points displays the heavy metal concentration distribution at a higher resolution with the caveat of having more uncertainty from heterogeneity. When relying on *on site* measurements, it is advisable to exercise some minimal preparation (Stephen Shefsky, 1997). Coarse samples should be lightly grinded as finer samples yield better results (Nuchdang et al., 2018). A preparation protocol could include the removal of debris, shaking the sample to achieve a degree of homogenization, filtering with a 2 mm sieve, and airdrying the sample in the sun. This procedure would decrease the usability and time savings of pXRF but increase the significance of each value, which would allow for a more reliable *on site* evaluation that could not be achieved just by measuring on the drill core in the conditions present at the Varta Insel, Hagen. Despite this, pXRF can help to determine which samples will be analyzed with conventional laboratory techniques and gives instant feedback during the field campaign.

### ICP-OES data versus pXRF data

**Fig. 9:**
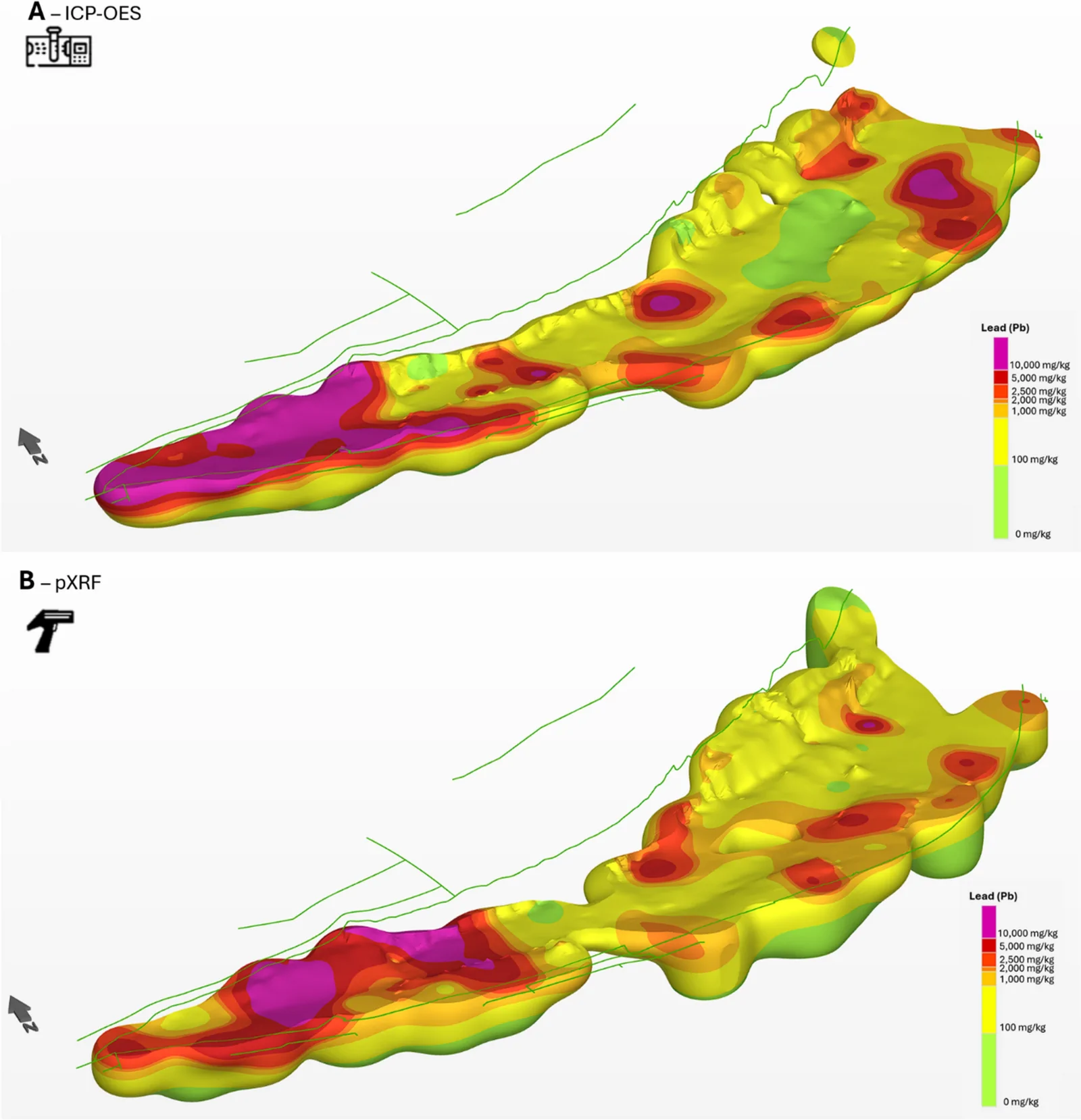
3D Model of the lead (Pb) concentration in the project area interpolated from pXRF (**B**) and ICP-OES (**A**) results. The large-scale distribution of lead is comparable in both models. Some high concentration regions are shaped differently i.e. on the western end of the area or along the southern boundary. Generally lower Pb concentrations can be observed on pXRF with some gaps of very low concentration near the middle. This 3D model was developed by Arcadis Germany GmbH on behalf of the Association for Land Recycling and Contaminated Site Remediation (AAV) using the same data as this study

**Fig. 10:**
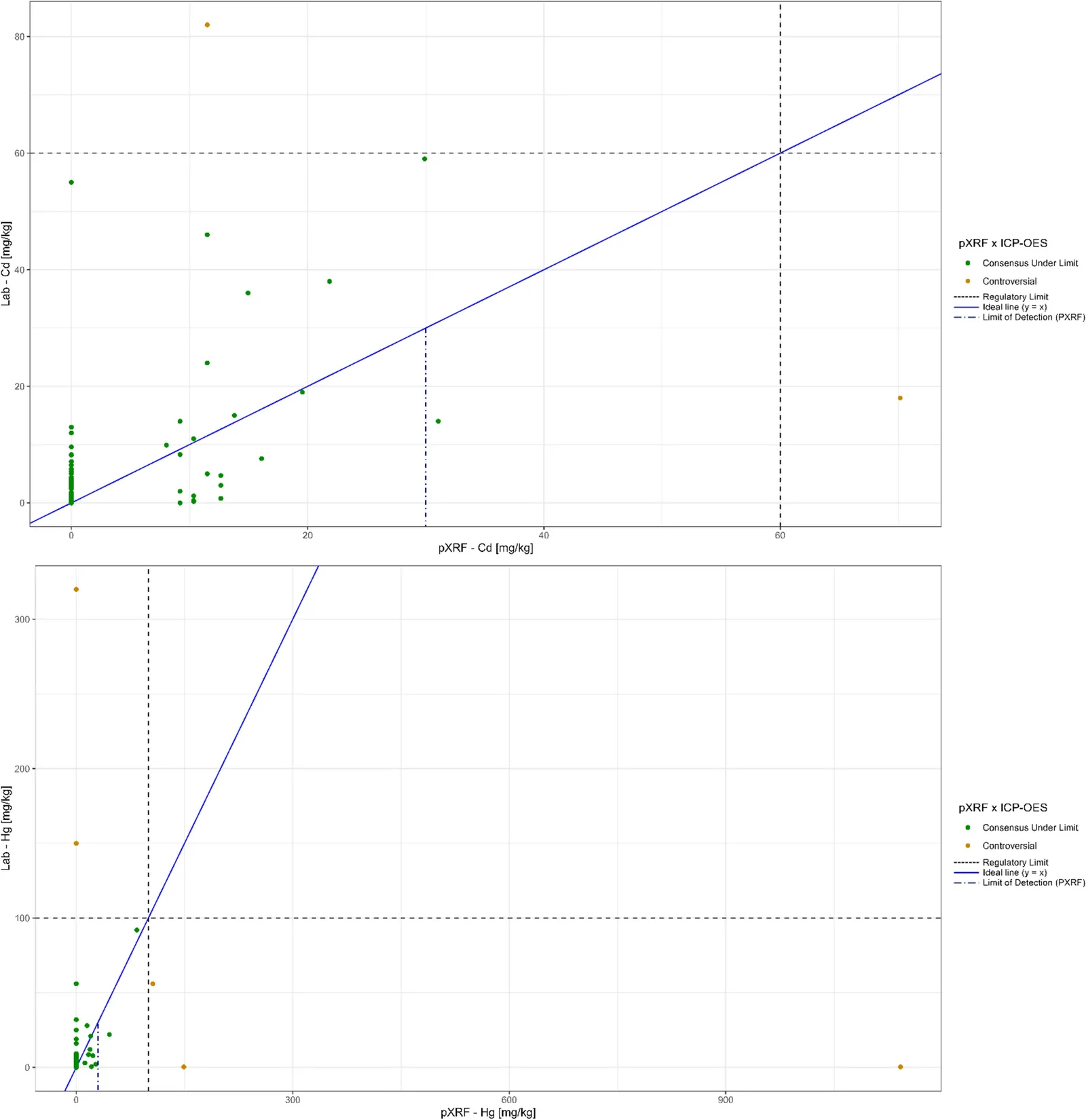
Direct comparison of the Cd and Hg laboratory results from ICP-OES and pXRF. Each sample with available ICP-OES (y-axis) data is plotted against its respective pXRF value (x-axis). The blue line (y = x) shows the ideal line, the dashed line represents the regulatory limit, the dot-dashed line shows the pXRF limit of detection. Most recorded Cd and Hg concentrations lay under the pXRF calibration zone. Thus, the results are not conclusive as to overall accuracy, but they are generally in agreement as to the regulatory limit

**Fig. 11:**
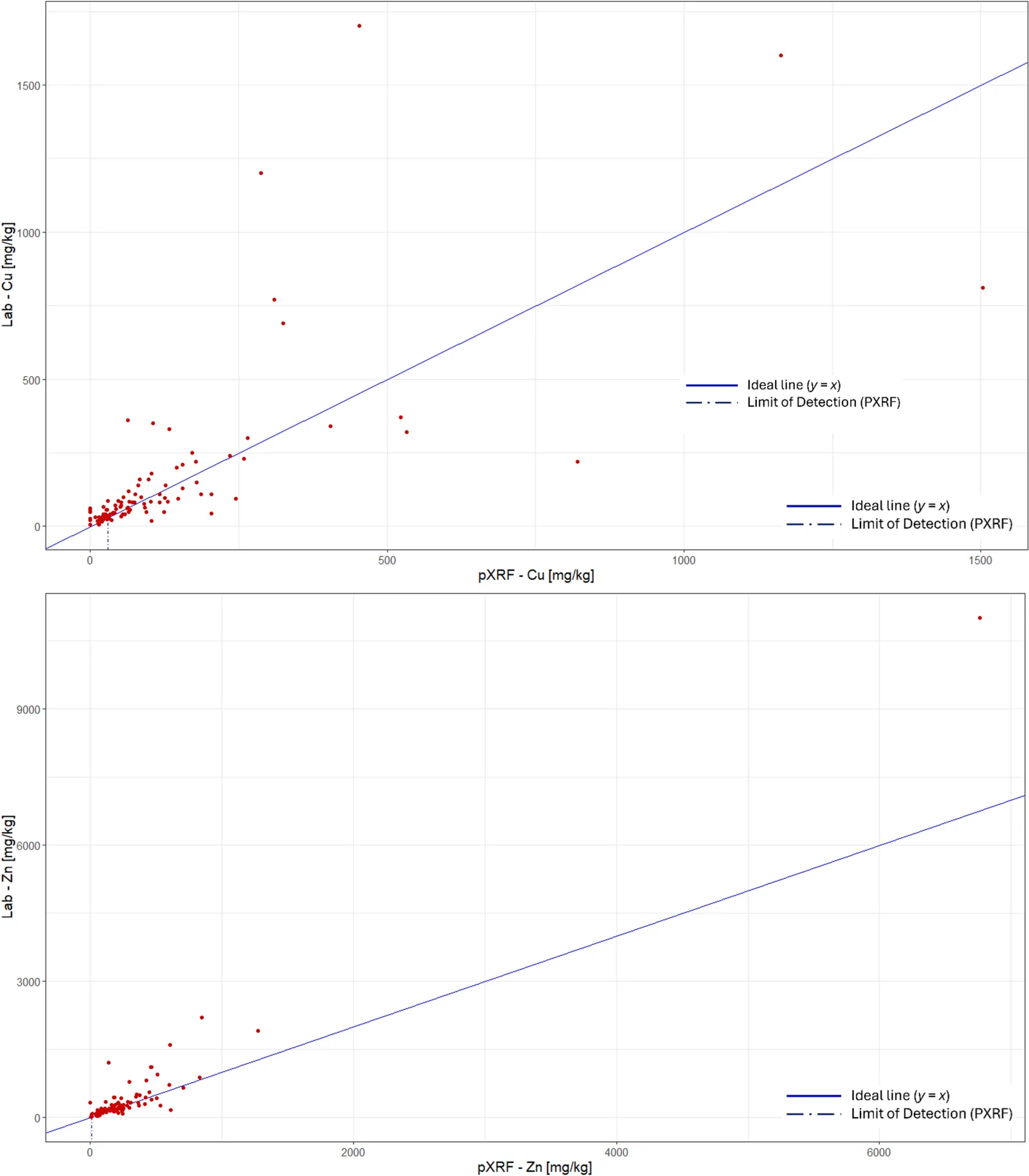
Direct comparison of the Cu and Zn laboratory results from ICP-OES and pXRF. Each sample with available ICP-OES (y-axis) data is plotted against its respective pXRF value (x-axis). The blue line (y = x) shows the ideal line, the dot-dashed line shows the pXRF limit of detection. There is no regulatory limit for Cu and Zn. Both the Cu and Zn results have a high consensus between pXRF and ICP-OES of the analyzed heavy metals. The Cu datapoints decrease in accuracy with higher concentration. There is no recognizable trend, pXRF gives comparable results to ICP-OES

**Fig. 12:**
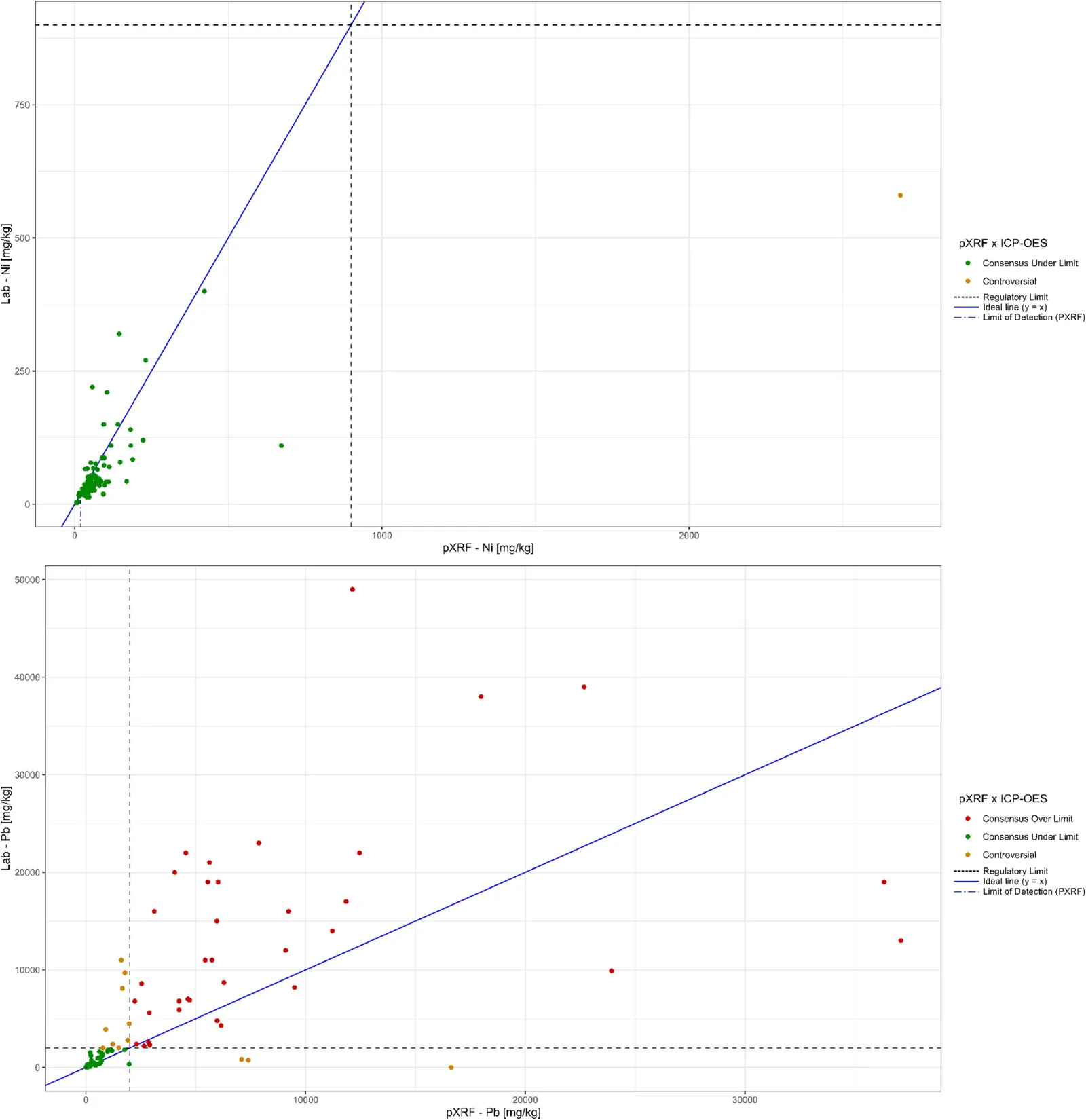
Direct comparison of the Ni and Pb laboratory results from ICP-OES and pXRF. Each sample with available ICP-OES (y-axis) data is plotted against its respective pXRF value (x-axis). The blue line (y = x) shows the ideal line, the dashed line represents the regulatory limit, the dot-dashed line shows the pXRF limit of detection. The Ni datapoints all plot close to each other showing good accordance between the pXRF and ICP-OES data. PXRF marginally overestimates the Ni concentration compared to ICP-OES. Contrary to this, pXRF systematically underestimates Pb concentration with larger deviations on high concentrations. Despite this almost all datapoints would lead to the same evaluation regarding the regulatory limits (Pb and Ni)

**Fig. 13:**
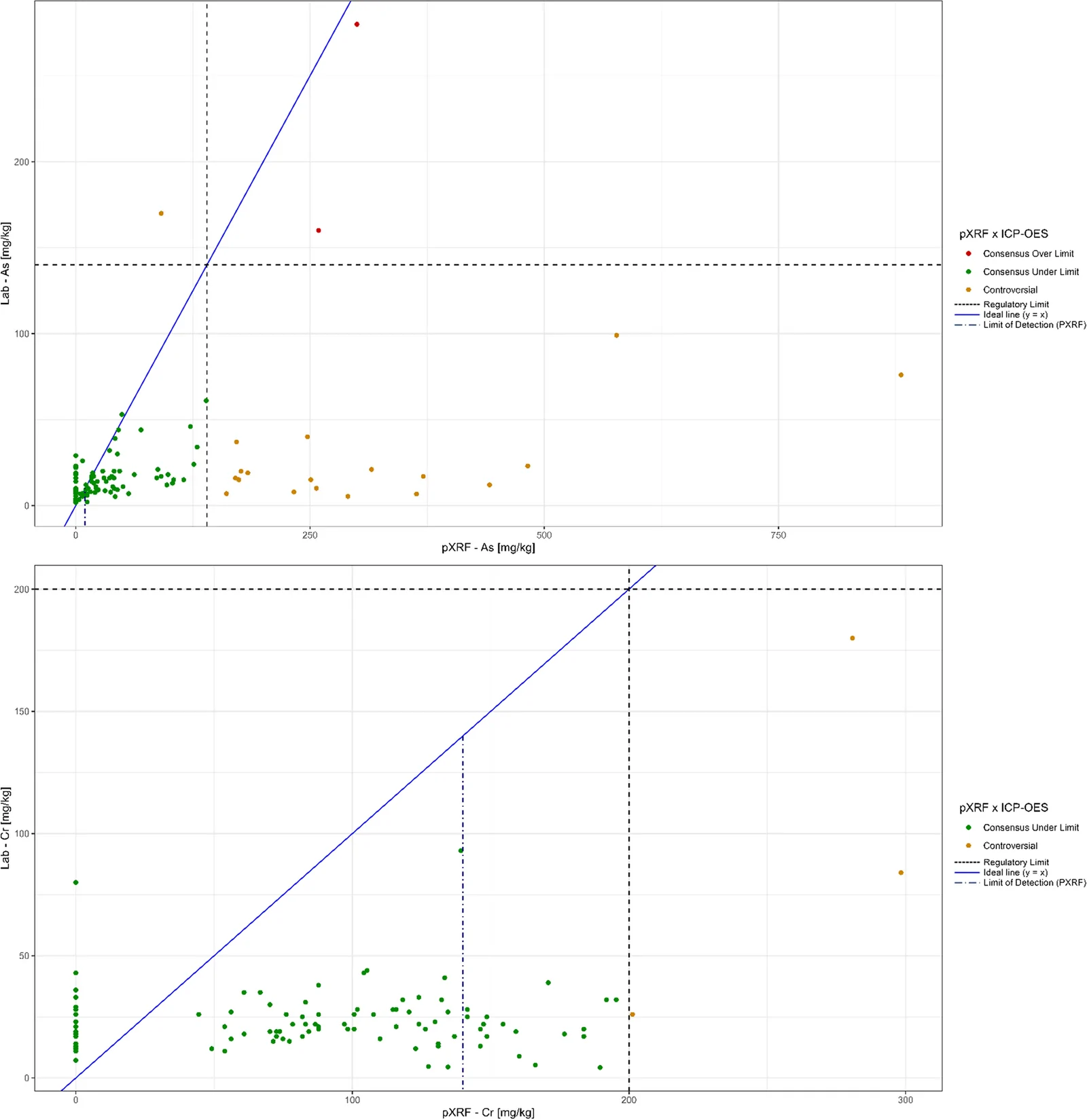
Direct comparison of the As and Cr laboratory results from ICP-OES and pXRF. Each sample with available ICP-OES (y-axis) data is plotted against its respective pXRF value (x-axis). The blue line (y = x) shows the ideal line, the dashed line represents the regulatory limit, the dot-dashed line shows the pXRF limit of detection. There is a systematic overestimation of As and Cr concentration from pXRF compared to ICP-OES. Most datapoints lie under the ideal line but have a consensus regarding the regulatory. The cause of the systematic error has to be further studied

To better understand the potential of pXRF, a comparison to the results of conventional wet chemistry like ICP-OES is needed. Since the samples originate from the same source, the resulting concentration from each sample and method can be directly compared (Figs. 9, 10, 11 and 12). Matching results plot near the ideal line, indicated as the blue 1:1 line for equal ICP-OES and pXRF concentrations (Figs. 9, 10, 11 and 12). The comparison of Cd and Hg is not conclusive as most measurements fall below the LOD of pXRF (Fig. 9). Yet, the results at lower concentrations below the LOD often show good agreement with the ICP-OES reference data. This suggests that the pXRF data in this range are more reliable than the nominal manufacture-provided LOD would imply. The results for Cu, Ni, and Zn are matching well with most datapoints close to the ideal line (Figs. 10 and 11). The absolute and relative difference between pXRF and ICP-OES increase with higher concentrations. There is no recognizable trend in the Cu, Ni, and Zn data, pXRF gives comparable results to ICP-OES. PXRF marginally overestimated Ni concentrations. For the elements As and Cr there was a systematic overestimation of concentrations in the pXRF results (Fig. 12). Another factor for the difference in concentration could be the incomplete aqua regia digestion. It should be considered that for As, ICP-OES measured a concentration above 100 mg/kg only 3 times while pXRF recorded 20 such values. In contrast, pXRF systematically underestimated Pb concentrations, with 6 compared to 14 recordings above 15,000 mg/kg. The systematic underestimation of Pb is particularly critical, as Pb is the most abundant heavy metal pollutant at this site. The cause of the systematic errors should be further studied. A 3D distribution model of Pb based on the collected pXRF and ICP-OES data was developed (Fig. 13, Source: Arcadis Germany GmbH, modified). The ICP-OES model contains results of previous external exploration campaigns from 2007 and 2017. The systematic underestimation of Pb by pXRF is apparent with overall lower concentrations. In the center region small areas of low Pb concentration appear only in the 3D model based on pXRF data (Fig. 13B). But the information on a larger scale and the resulting highly probable polluted areas are similar in both models. How do the differences in heavy metal concentrations translate into correctly evaluating soil based on regulatory levels? Table 1 shows the maximum allowed concentrations permitted by German regulatory limits for the effective pathway from soil to humans in industrial and commercial areas. For Cu and Zn there is no regulatory limit in this category. Comparing the pXRF and ICP-OES recorded concentrations results in a total of 12 false negatives, 27 false positives, 46 consensus positives and 548 consensus negatives. In almost 94% of the cases, the two methods come to the same conclusion. A false positive means that the concentration measured by pXRF exceeds the regulatory level while the same sample measured by ICP-OES resulted in a concentration below the regulatory level, and vice versa. Consensus means that the two methods both show concentrations below or above the regulatory limit. This assumes that the ICP-OES values are correct.

**Table 1:** German regulatory limits for industrial and commercial areas (Path: Soil—Human, Germany, BBodSchV, BGBl 2021)

Element	Antimony	Arsenic	Cadmium	Chromium	Mercury	Nickel	Lead
[mg/kg]	250	140	60	200	100	900	2000

The systematic overestimation of As and Cr could also result from incomplete aqua regia digestion of As- and Cr-rich components. It cannot be ruled out that a complete digestion of the soil material would have yielded a higher degree of agreement. Errors from sample preparation could affect the pXRF results as well. Insufficient homogenization of the sample could have produced variable concentrations and reduced overall reliability. Additional grinding or even pressing the soil into pellets could improve the overall accuracy of pXRF (Goff et al., 2020). Developing optimized operating modes and using reference materials representative of urban soils could further improve the reliability of pXRF in remediation projects. Nevertheless, we want to highlight that pXRF correctly classified ~94% of samples compared to ICP-OES. While only 106 of 230 samples were analyzed conventionally with ICP-OES, the much higher data density of pXRF provides broader insight and supports targeted selection of samples for laboratory analysis despite lower analytical accuracy.

### Application of pXRF in remediation projects

Although pXRF results can show high precision but variable accuracy, its specific advantages make it a valuable tool for remediation projects. Cost and time savings are significant, but potential biases from sampling bags, grain size, moisture content, and soil heterogeneity must be carefully controlled. This study demonstrated that in-situ field results, measured directly on unprepared soil material within ram cores, provide first-order indications of heavy metal contamination. In addition, the results from samples in plastic bags that were dried until no more moisture was visible yield the same classification as ICP-OES in 94% of the time.

In the remediation project Varta Insel, Hagen, less than half of the samples were analyzed by the accredited laboratory while all samples were measured by pXRF. This puts a lot of weight on each data point and increases the potential for sampling bias. In addition to the eight heavy metals tested by default in the ICP-OES data set, each pXRF measurement recorded the concentration of 43 elements. This led to the discovery of Sb in certain project areas which might have gone undetected without the use of pXRF, as Sb is not routinely analyzed in remediation projects by ICP-OES. An overall larger number of data points with more geochemical information leads to a better understanding of the contaminated site than a small number of data points with greater accuracy and confidence.

Lemière (2018) suggested that the best results can be achieved through a clever combination of *on site* and conventional laboratory data, with pXRF providing the bulk of the data at low cost, and cross monitoring using reliable lab-based analysis on a limited number of samples. The pXRF data helped identify which samples to analyze at Varta Insel, Hagen in addition to providing valuable information for the 3D distribution model (Fig. 9). The presented results indicate that the advantages outweigh the limitations of using pXRF in remediation projects.

Despite the promising results, the observed systematic errors on Pb, As, and Cr as well as the high LOD close to regulatory levels for Cr and Cd have to be considered when using pXRF for remediation projects. Inconsistencies between results from the applied methods meant that some samples such as from sampling point 34, could only be used for a semi-quantitative assessment and require further testing. By establishing a standard operating procedure, the obstacles of using the technique could be reduced to a minimum.

## Conclusion and outlook

This study demonstrates the potential of pXRF for assessing heavy-metal-contaminated soils, both on-site without any sample preparation, and under laboratory conditions. Measuring soil samples by pXRF through plastic bags does influence concentrations, but establishing empirical correction factors can mitigate this effect. Hence, measuring contaminated soil samples through LDPE sample bags becomes a practical option for rapid and cost-efficient analysis.

Laboratory pXRF results corresponded with ICP-OES classifications in ~94% of cases and additionally revealed Sb contamination, which would have remained undetected by ICP-OES only. The laboratory pXRF data moreover also provided a dense sampling grid, allowing for 3D modeling of contamination geometries. However, pXRF has limitations and should not be considered a stand-alone substitute for conventional laboratory analysis. Under laboratory conditions, there were systemic deviations: Pb was underestimated, whereas As and Cr were overestimated relative to ICP-OES.

On-site drill-core measurements yielded higher resolution but greater uncertainty from moisture and limited sample preparation, highlighting the need for targeted laboratory validation. Sampling error remains the main source of uncertainty, but large pXRF datasets combined with selective wet-chemistry analyses offer a cost-effective approach. Future studies should quantify moisture effects, test simple preparation methods, and explore applications such as measuring heavy metals in surrounding plants to assess ecotoxicological impacts.

## Supplementary Information

Below is the link to the electronic supplementary material.

Supplementary file1 (DOCX 311 KB)

Supplementary file2 (PDF 545 KB)

## Data Availability

Data will be made available if requested.
