# Supplementary material for: Portable X-ray fluorescence (pXRF) analysis of heavy metals in contaminated soils: a case study from Varta Insel, Hagen
Source: Environ Geochem Health. 2026 Oct 27;48(16):667. doi: 10.1007/s10653-026-03538-5 (PMC13714078; doi:10.1007/s10653-026-03538-5)
Supplement: Supplementary file 1 [file 10653_2026_3538_MOESM2_ESM.pdf]

SM.5 Plastic Bag Experiment Results

sample	MgO	Al_2O_3	SiO_2	P	S	Cl	K_2O	Ca	Ti	V	Cr	Mn	Fe	Ni
CS-M2 standard with plastic bag -1	0,5416	0,7782	10,3417	0	0,0225	0,0212	2,3454	0,1473	0	0,0087	0	0,1321	1,5143	0,0049
CS-M2 standard with plastic bag -2	0,5197	0,8016	10,761	0	0,0409	0,0414	2,3771	0,1766	0	0,0065	0	0,1341	1,5419	0,0047
CS-M2 standard with plastic bag -3	0,6727	0,8969	10,7982	0	0,0236	0	2,3704	0,1664	0	0	0	0,133	1,5369	0,0047
CS-M2 standard with plastic bag -4	0,4549	0,9599	11,7415	0	0,0279	0,0255	2,4414	0,1802	0	0,0115	0	0,1304	1,5567	0,0048
CS-M2 standard with plastic bag -5	0,5303	0,889	11,5678	0	0,0203	0	2,3715	0,1736	0	0,0071	0	0,1367	1,5042	0,0047
CS-M2 standard with plastic bag -6	0,7024	0,881	11,3653	0	0,0323	0,032	2,3676	0,1788	0	0,0062	0	0,1328	1,5192	0,0051
CS-M2 standard with plastic bag -7	0,361	0,8199	11,6384	0	0,0336	0,024	2,4324	0,1813	0	0	0	0,1331	1,5627	0,0046
CS-M2 standard with plastic bag -8	0,723	0,8311	11,694	0	0,0279	0,045	2,4343	0,1868	0	0	0	0,1394	1,567	0,0049
CS-M2 standard with plastic bag -9	0	0,7609	10,5276	0	0,0323	0,0202	2,3193	0,1441	0	0,0059	0	0,1276	1,489	0,0046
CS-M2 standard with plastic bag -10	0,4734	0,8023	10,8243	0	0,0271	0,0518	2,3663	0,1573	0	0	0,0061	0,1371	1,5301	0,0042
CS-M2 standard with plastic bag -11	0,5974	0,8345	11,4482	0	0,0274	0	2,3173	0,158	0	0,0114	0	0,1284	1,4523	0,0051
CS-M2 standard -1	0,5194	9,8309	67,8171	0	0,1938	0	4,1299	0,5389	0,1307	0,0075	0,004	0,1005	1,5577	0,0044
CS-M2 standard -2	0,4868	9,6299	67,5063	0	0,2119	0	4,079	0,5288	0,1257	0	0	0,096	1,5286	0,0041
CS-M2 standard -3	0,5163	10,0156	67,949	0	0,2304	0	4,0939	0,5305	0,1261	0	0,0043	0,0945	1,5068	0,0045
CS-M2 standard -4	0,4401	9,5114	67,2127	0	0,2237	0	3,9958	0,5298	0,1222	0	0,0048	0,0953	1,4732	0,0046
CS-M2 standard -5	0	9,5378	66,4444	0	0,2044	0	3,9518	0,5237	0,1242	0	0,0045	0,0981	1,4633	0,0043
CS-M2 standard -6	0	9,8056	68,2	0	0,2187	0	4,1151	0,5281	0,1232	0	0,0099	0,0981	1,5146	0,0046
CS-M2 standard -7	0,4499	10,0654	68,9668	0	0,2231	0	4,1283	0,5412	0,1278	0,0062	0,0057	0,0991	1,5159	0,0044
CS-M2 standard -8	0,6544	10,0023	68,8715	0,0131	0,2439	0	4,1345	0,5455	0,1286	0	0,0052	0,0989	1,5194	0,0049
CS-M2 standard -9	0	9,7955	68,5187	0	0,2368	0	4,0562	0,5194	0,1305	0	0,0065	0,0975	1,506	0,005
CS-M2 standard -10	0,3857	10,0433	68,7285	0,0157	0,2544	0	4,0969	0,5359	0,1321	0	0,0099	0,0997	1,5213	0,0059
CS-M2 standard -11	0,5875	9,8742	68,1814	0	0,204	0	4,2809	0,5536	0,1313	0	0	0,0995	1,5838	0,0048
CS-M2 standard -12	0,5465	9,3831	65,4765	0	0,2095	0	4,0783	0,5263	0,1248	0	0,014	0,0997	1,5423	0,0049
JMS-2 standard -1	1,8935	9,336	35,6016	0,3767	0,2034	3,0092	1,6713	2,5268	0,6269	0,0132	0	1,6522	6,2107	0,0309
JMS-2 standard -2	2,4457	9,2016	35,7331	0,3737	0,2107	3,0569	1,6846	2,5342	0,6384	0	0	1,6686	6,2627	0,0285
JMS-2 standard -3	2,2241	9,5778	36,9002	0,3671	0,2108	3,1033	1,7072	2,5704	0,6459	0,0121	0	1,6803	6,2697	0,0304
JMS-2 standard -4	2,2117	9,7212	37,1093	0,3782	0,2119	3,1349	1,7272	2,5672	0,6387	0,0089	0	1,655	6,2719	0,0312
JMS-2 standard -5	2,0652	9,4379	36,85	0,3588	0,2055	3,1333	1,7275	2,5569	0,6394	0,0104	0	1,6835	6,2801	0,0294
JMS-2 standard -6	2,0144	9,7984	37,5486	0,3798	0,2185	3,1318	1,7473	2,5971	0,6401	0,0128	0	1,6625	6,3235	0,0316
JMS-2 standard -7	2,3331	9,8055	37,4127	0,3895	0,2239	3,1444	1,7363	2,5818	0,6406	0,0139	0	1,6569	6,2983	0,0313
JMS-2 standard -8	1,9086	9,7129	38,0131	0,388	0,2237	3,208	1,748	2,5839	0,641	0	0	1,6886	6,3099	0,0304
JMS-2 standard -9	2,3392	9,9707	38,5659	0,3935	0,2211	3,2131	1,7718	2,6195	0,6477	0	0	1,6641	6,3439	0,0297
JMS-2 standard -10	2,4031	9,4311	36,8798	0,3832	0,2122	3,1047	1,7281	2,558	0,6405	0	0	1,6543	6,2588	0,03
JMS-2 standard with bag -1	0,7732	0,906	5,2955	0,0812	0,0174	1,5325	1,043	1,8706	0,3987	0	0	1,922	5,9381	0,0285
JMS-2 standard with bag -2	0,7965	0,8659	5,6263	0,0903	0,0215	1,6627	1,096	1,9288	0,4446	0	0	1,909	5,9404	0,0293
JMS-2 standard with bag -3	0,7844	0,8717	4,7152	0,0751	0,0105	1,4288	0,9876	1,786	0,4058	0	0	1,9076	5,7422	0,0302
JMS-2 standard with bag -4	0,625	0,7323	4,5552	0,0733	0	1,41	0,9792	1,7811	0,3814	0	0	1,8963	5,677	0,0296
JMS-2 standard with bag -5	0,7125	0,8094	4,0328	0,0664	0,0173	1,4337	1,0053	1,8268	0,3714	0	0	1,9061	5,7104	0,0279
JMS-2 standard with bag -6	0,4868	0,5791	3,6316	0,0575	0	1,3014	0,9005	1,609	0,2879	0	0	1,7611	5,1514	0,028
JMS-2 standard with bag -7	0,7049	0,7057	3,8566	0,0618	0	1,2877	0,9404	1,6775	0,3351	0	0	1,8358	5,4299	0,029
SARM 46 standard -1	1,9002	6,62	37,4612	0,0209	0,4038	0	0,3068	1,0243	0,2766	0	0,0659	1,0017	19,9074	0,0262
SARM 46 standard -2	1,8428	6,5972	37,5729	0,0185	0,3873	0	0,3025	1,0256	0,2799	0,0076	0,0572	1,0075	19,858	0,0256
SARM 46 standard -3	1,553	6,7333	37,7469	0,0164	0,4013	0	0,3064	1,0303	0,2821	0,0103	0,0628	1,0081	19,897	0,0261
SARM 46 standard -4	2,2896	7,0573	37,8614	0,0272	0,408	0	0,3167	1,0507	0,2872	0,0067	0,064	1,0161	19,9003	0,0289
SARM 46 standard -5	1,932	7,0683	37,4635	0,0175	0,3963	0	0,3137	1,0483	0,2902	0	0,0584	1,0076	19,8138	0,0264
SARM 46 standard -6	1,9961	7,0172	37,5842	0,0297	0,3879	0	0,3217	1,0505	0,2912	0,0096	0,0596	0,9922	19,8778	0,0278
SARM 46 standard -7	2,5153	6,9921	38,1151	0,0212	0,4027	0	0,3236	1,0531	0,2929	0	0,0567	1,0103	20,0164	0,0267
SARM 46 standard -8	2,163	7,0546	38,0107	0,0204	0,3928	0	0,2972	1,0386	0,2869	0,0151	0,0699	1,0176	19,9982	0,0269
SARM 46 standard -9	1,82	6,9523	38,1846	0,0179	0,414	0	0,3255	1,0633	0,2846	0	0,0567	1,0088	19,9315	0,0239

SM.5 Plastic Bag Experiment Results

sample	MgO	Al2O3	SiO2	P	S	Cl	K2O	Ca	Ti	V	Cr	Mn	Fe	Ni
SARM 46 standard -10	2,057	7,0482	38,4779	0	0,3856	0	0,3195	1,0553	0,2879	0	0,0522	0,9922	19,8147	0,0231
SARM 46 standard with bag -1	0,7603	0,6112	4,9023	0	0	0,0282	0,1969	0,7428	0,2354	0	0,0541	1,1341	19,0998	0,0234
SARM 46 standard with bag -2	0,9863	0,6607	4,5536	0,0109	0	0,0294	0,1788	0,7225	0,2313	0	0,0516	1,0999	18,2689	0,0244
SARM 46 standard with bag -3	0,9093	0,6034	3,9233	0	0	0,0419	0,1807	0,8052	0,2248	0	0,0528	1,1582	19,2281	0,0235
SARM 46 standard with bag -4	0,675	0,5684	4,0573	0	0	0	0,1881	0,7359	0,2346	0	0,0532	1,1311	18,5302	0,0184
SARM 46 standard with bag -5	0,9296	0,6649	4,7004	0	0	0,0241	0,1794	0,735	0,2397	0	0,0423	1,1196	18,8666	0,0223
SARM 46 standard with bag -6	0,6482	0,6034	4,239	0	0	0	0,1598	0,6744	0,2112	0	0,0528	1,0745	17,554	0,0244
SARM 46 standard with bag -7	0,7983	0,5817	4,4592	0	0	0	0,1606	0,6614	0,2161	0	0,0433	1,0345	17,0095	0,0241
SARM 46 standard with bag -8	0,9485	0,6621	4,1026	0	0	0	0,1842	0,7251	0,2258	0	0,06	1,1381	19,0618	0,0229
SARM 46 standard with bag -9	1,1888	0,5598	3,8292	0	0	0	0,1661	0,6925	0,23	0	0,0552	1,0947	17,8485	0,0236
SARM 46 standard with bag -10	0,8858	0,5964	4,1664	0	0	0	0,1835	0,7173	0,2332	0	0,0503	1,122	18,8652	0,024
CS-M2 Target value	NA	10,53	NA	NA	NA	NA	4,36	0,55	NA	NA	NA	0,102	1,59	NA
CS-M2 - Mean	0,38221667	9,79125	67,8227417	0,0024	0,221216667	0	4,09505	0,533475	0,12726667	0,00114167	0,00573333	0,098075	1,51940833	0,0047
Standard Deviation	0,23042745	0,21935748	0,99487915	0,00539274	0,017112025	0	0,07705083	0,00941117	0,00323325	0,0025666	0,00384195	0,00182671	0,03146714	0,00044535
% change to Mean	0,00602871	0,00022403	0,00014669	0,02246976	0,000773541	0	0,00018816	0,00017641	0,00025405	0,02248116	0,00670107	0,00018626	0,0002071	0,00094755
CS-M2 with Bag - Mean	0,50694545	0,84139091	11,1552727	0	0,028709091	0,02373636	2,37663636	0,16821818	0	0,00520909	0,00055455	0,13315455	1,52493636	0,00475455
Standard Deviation	0,19178006	0,05653616	0,48764429	0	0,005553943	0,01740524	0,04119462	0,01387397	0	0,00432045	0,00175363	0,00344235	0,03276004	0,00024258
% change to Mean	0,00378305	0,00067194	0,00043714	0	0,001934559	0,00733273	0,00017333	0,00082476	0	0,00829406	0,03162278	0,00025852	0,00021483	0,0005102
Factor	0,75396014	11,636981	6,07988198	0	7,70545704	0	1,72304441	3,17132782	0	0,21916812	10,3387978	0,73655015	0,99637491	0,98852772
Standards Target value														
JMS-2 published values	3,24	14,18	41,78	0,54936	0,3085	4,05	2,7	4,68	0,83902	0,0183	0,0078	1,74924	7,665424	0,0311
JMS-2 - Mean	2,18386	9,59931	37,06143	0,37885	0,21417	3,12396	1,72493	2,56958	0,63992	0,00713	0	1,6666	6,28295	0,03034
JMS-2 with Bag - Mean	0,69761429	0,78144286	4,53045714	0,07222857	0,009528571	1,43668571	0,99314286	1,78282857	0,37498571	0	0	1,87684286	5,65562857	0,02892857
Factor	3,13046915	12,2840844	8,18050559	5,24515427	22,47661169	2,17442129	1,73683976	1,44129393	1,70651834	0	0	0,88798058	1,11091984	1,04879012
SARM 46 published values	3,16	6,71	35,9	0,04796	0,17	0	0,35	0,943404	0,35958	0,0225	0,0559	0,88236	19,8	0,0127
SARM 46 - Mean	2,0069	6,91405	37,84784	0,01897	0,39797	0	0,31336	1,044	0,28595	0,00493	0,06034	1,00621	19,90151	0,02616
Standard Deviation	0,25500279	0,1788264	0,3257319	0,00750734	0,009070838	0	0,00917237	0,01277545	0,0049078	0,0053466	0,00498682	0,00819176	0,06417855	0,00160885
% change to Mean	0,00127063	0,00025864	8,6064E-05	0,00395748	0,000227928	0	0,00029271	0,00012237	0,00017163	0,01084502	0,00082645	8,1412E-05	3,2248E-05	0,000615
SARM 46 with Bag - Mean	0,87301	0,6112	4,29333	0,00109	0	0,01236	0,17781	0,72121	0,22821	0	0,05156	1,11067	18,43326	0,0231
Standard Deviation	0,15197773	0,03689108	0,33095222	0,00327	0	0,0157109	0,01145884	0,03814599	0,00845949	0	0,00502259	0,0341572	0,70880258	0,00168701
% change to Mean	0,00174085	0,00060358	0,00077085	0,03	0	0,01271108	0,00064444	0,00052892	0,00037069	0	0,00097413	0,00030754	0,00038452	0,00073031
Factor	2,29882819	11,3122546	8,81549753	17,4036697	0	0	1,76233058	1,44756728	1,25301258	0	1,17028704	0,90594866	1,07965222	1,13246753
Limit of Detection	0,5	0,12		0,015	0,03	0,025	0,016	0,01	0,01	0,008	0,014	0,005	0,005	0,002
Upper Limit	70	69	100	13,2	3,7	4	15,4	31,1	19,4	10,7	2,4	16	34,5	1,4

all values in mg/kg

SM.5 Plastic Bag Experiment Results

sample	Cu	Zn	Ga	As	Se	Rb	Sr	Y	Zr	Nb	Mo	Ag	Cd	Sn
CS-M2 standard with plastic bag -1	0,0158	0,0451	0,0012	0,0059	0	0,0123	0,0233	0,0077	0,0634	0,0014	0	0	0	0
CS-M2 standard with plastic bag -2	0,0161	0,0434	0,0005	0,004	0	0,0125	0,0223	0,0084	0,0679	0,0016	0	0	0	0
CS-M2 standard with plastic bag -3	0,0162	0,0445	0,0008	0,0056	0,0004	0,012	0,0229	0,0079	0,0643	0,0015	0	0	0	0
CS-M2 standard with plastic bag -4	0,0153	0,0453	0	0,0052	0	0,0119	0,0231	0,008	0,0689	0,0012	0	0	0	0
CS-M2 standard with plastic bag -5	0,0161	0,044	0,0005	0,0062	0	0,0123	0,022	0,0079	0,0585	0,0016	0	0	0	0
CS-M2 standard with plastic bag -6	0,0155	0,0437	0,001	0,0059	0	0,012	0,0232	0,0081	0,0643	0,0021	0	0	0	0
CS-M2 standard with plastic bag -7	0,0158	0,0438	0,0009	0,0065	0,0005	0,0125	0,0233	0,0081	0,0636	0,0019	0	0	0,001	0
CS-M2 standard with plastic bag -8	0,0161	0,0456	0,0007	0,0056	0	0,0121	0,0224	0,008	0,0711	0,0017	0	0	0	0
CS-M2 standard with plastic bag -9	0,0157	0,0427	0,001	0,0053	0,0003	0,0118	0,0227	0,0084	0,0656	0,002	0	0	0,0012	0
CS-M2 standard with plastic bag -10	0,0159	0,0448	0,0009	0,0054	0	0,0118	0,0232	0,0074	0,0654	0,0019	0	0	0	0
CS-M2 standard with plastic bag -11	0,0159	0,0457	0,0006	0,0069	0	0,0118	0,0217	0,0073	0,0596	0,002	0	0	0	0
CS-M2 standard -1	0,0195	0,0638	0,0014	0,0079	0	0,0129	0,0135	0,0038	0,0328	0,0013	0	0	0	0
CS-M2 standard -2	0,0197	0,0654	0,0012	0,0058	0	0,0129	0,0132	0,0037	0,0333	0,001	0	0	0	0
CS-M2 standard -3	0,0188	0,0637	0,0015	0,0049	0,0003	0,0124	0,0131	0,0036	0,0315	0,0015	0	0	0	0
CS-M2 standard -4	0,0196	0,062	0,0009	0,0064	0	0,0125	0,0133	0,0035	0,0323	0,001	0	0	0	0
CS-M2 standard -5	0,0199	0,0634	0,0013	0,006	0,0003	0,0124	0,0135	0,0035	0,0338	0,0011	0	0	0	0
CS-M2 standard -6	0,0198	0,0636	0,001	0,0057	0	0,0121	0,0135	0,0034	0,0329	0,0016	0	0	0	0
CS-M2 standard -7	0,0193	0,0654	0,001	0,0058	0	0,0122	0,0133	0,0031	0,0305	0,0017	0	0	0	0
CS-M2 standard -8	0,0197	0,0637	0,0013	0,0055	0	0,0123	0,0131	0,0032	0,0318	0,0013	0	0	0	0
CS-M2 standard -9	0,0194	0,062	0,001	0,0045	0	0,0121	0,0135	0,0036	0,0312	0,0011	0	0	0	0
CS-M2 standard -10	0,0186	0,0612	0,0013	0,0055	0,0005	0,0123	0,0136	0,0037	0,0318	0,0009	0	0	0	0
CS-M2 standard -11	0,0199	0,0651	0,001	0,0064	0	0,0125	0,0139	0,0038	0,0329	0,0013	0	0	0	0
CS-M2 standard -12	0,0194	0,0625	0,0017	0,0049	0	0,0127	0,0142	0,0037	0,0348	0,0013	0	0	0,001	0
JMS-2 standard -1	0,0367	0,012	0,001	0,0039	0	0,0057	0,0387	0,0243	0,0246	0	0	0	0	0
JMS-2 standard -2	0,0381	0,012	0,0011	0,0024	0	0,0059	0,0396	0,025	0,0252	0	0	0	0	0
JMS-2 standard -3	0,0369	0,0115	0,0006	0,0028	0	0,0058	0,0382	0,0239	0,0239	0	0	0	0	0
JMS-2 standard -4	0,0366	0,0118	0,001	0,0035	0	0,0057	0,0387	0,0239	0,0243	0	0	0	0	0
JMS-2 standard -5	0,0374	0,011	0,0009	0,0036	0	0,0053	0,0384	0,0247	0,0248	0	0	0	0	0
JMS-2 standard -6	0,0365	0,0116	0,0006	0,0034	0	0,0059	0,0404	0,0239	0,0243	0	0	0	0	0
JMS-2 standard -7	0,0366	0,0122	0,001	0,0028	0	0,0057	0,0393	0,0249	0,025	0	0	0	0,0009	0
JMS-2 standard -8	0,0381	0,0109	0,0009	0,0027	0	0,0063	0,0386	0,0241	0,0259	0	0,0011	0	0	0
JMS-2 standard -9	0,0362	0,0122	0,0011	0,0024	0	0,006	0,0401	0,0243	0,0244	0	0,0013	0	0	0
JMS-2 standard -10	0,0375	0,0114	0,001	0,0029	0	0,0059	0,0393	0,0238	0,0244	0	0	0	0	0
JMS-2 standard with bag -1	0,032	0,0087	0,0008	0,0025	0	0,0056	0,0477	0,0352	0,0285	0	0,0019	0	0	0
JMS-2 standard with bag -2	0,0327	0,0094	0,0007	0,0031	0	0,0051	0,0471	0,0342	0,0284	0	0,002	0	0	0
JMS-2 standard with bag -3	0,0321	0,0096	0	0,0032	0	0,0058	0,0474	0,0343	0,0246	0	0	0	0	0
JMS-2 standard with bag -4	0,0316	0,009	0	0,003	0	0,0058	0,0462	0,0336	0,0255	0	0	0	0	0
JMS-2 standard with bag -5	0,0312	0,0085	0,0006	0,0022	0	0,0058	0,0478	0,0344	0,0296	0	0	0	0	0
JMS-2 standard with bag -6	0,031	0,0082	0,0009	0,002	0	0,005	0,0434	0,0314	0,0225	0	0,0012	0	0,0007	0
JMS-2 standard with bag -7	0,0307	0,0089	0,0012	0,0026	0	0,0054	0,0437	0,0319	0,0251	0	0,0013	0	0	0
SARM 46 standard -1	0,0401	0,5986	0,0023	0,0781	0	0,0039	0,0044	0,0024	0,0053	0	0	0,0038	0,002	0
SARM 46 standard -2	0,0407	0,582	0,002	0,0896	0	0,004	0,0041	0,0026	0,0057	0	0	0,0032	0,0027	0
SARM 46 standard -3	0,0438	0,6065	0	0,0797	0	0,0041	0,0038	0,003	0,0057	0	0	0,0034	0,0018	0
SARM 46 standard -4	0,0376	0,6057	0	0,0755	0	0,0032	0,0038	0,0018	0,0047	0	0	0,0036	0,003	0
SARM 46 standard -5	0,0412	0,6028	0	0,0845	0	0,0037	0,0041	0,0029	0,0056	0	0	0,0038	0,0028	0,003
SARM 46 standard -6	0,0397	0,5974	0	0,0866	0	0,0032	0,0038	0,0025	0,0048	0	0	0,0048	0,0023	0,0035
SARM 46 standard -7	0,0413	0,6039	0,0028	0,0819	0	0,0034	0,0038	0,0018	0,0046	0	0	0,0036	0,002	0
SARM 46 standard -8	0,0394	0,5931	0,0032	0,0951	0	0,0036	0,0035	0,0023	0,005	0	0	0,0039	0,0024	0
SARM 46 standard -9	0,0417	0,5981	0,0021	0,0791	0	0,0039	0,0035	0,0027	0,0052	0	0	0,0035	0,0025	0

SM.5 Plastic Bag Experiment Results

sample	Cu	Zn	Ga	As	Se	Rb	Sr	Y	Zr	Nb	Mo	Ag	Cd	Sn
SARM 46 standard -10	0,0388	0,5944	0,002	0,0806	0	0,0034	0,0038	0,0021	0,005	0	0	0,0036	0,0033	0
SARM 46 standard with bag -1	0,0325	0,4456	0	0,0786	0	0,003	0,004	0,0031	0,0105	0	0	0,0024	0,0019	0
SARM 46 standard with bag -2	0,0329	0,4347	0	0,0624	0	0,0032	0,0046	0,0043	0,0097	0	0	0,0039	0,0021	0
SARM 46 standard with bag -3	0,0352	0,4498	0	0,0657	0	0,0026	0,0047	0,0031	0,0127	0	0	0	0,002	0
SARM 46 standard with bag -4	0,0265	0,3497	0	0,0569	0	0,0023	0,0041	0,0038	0,0118	0	0,0015	0	0,0012	0
SARM 46 standard with bag -5	0,0306	0,445	0	0,0755	0	0,0035	0,0044	0,0028	0,0125	0	0	0,0032	0,0024	0
SARM 46 standard with bag -6	0,0338	0,4233	0	0,063	0,0004	0,0025	0,004	0,003	0,0124	0	0	0,0031	0,0026	0
SARM 46 standard with bag -7	0,0307	0,4335	0	0,0678	0	0,0026	0,0037	0,0027	0,011	0	0	0,0028	0,0028	0
SARM 46 standard with bag -8	0,0338	0,429	0	0,0658	0	0,0035	0,0046	0,004	0,0116	0	0	0,0037	0,0023	0
SARM 46 standard with bag -9	0,0325	0,419	0	0,0658	0	0,0029	0,0035	0,0031	0,0122	0	0	0,0029	0,0018	0
SARM 46 standard with bag -10	0,0301	0,4345	0	0,0753	0	0,0027	0,0038	0,0038	0,0106	0	0	0,0032	0,0024	0
CS-M2 Target value	0,02	NA	NA	NA	NA	NA	NA	NA	NA	NA	NA	NA	NA	NA
CS-M2 - Mean	0,01946667	0,06348333	0,00121667	0,005775	9,16667E-05	0,01244167	0,013475	0,00355	0,03246667	0,00125833	0	0	8,3333E-05	0
Standard Deviation	0,00039229	0,00131519	0,00023393	0,00084963	0,000165622	0,00026286	0,00030856	0,00021409	0,00114479	0,00023965	0	0	0,00027639	0
% change to Mean	0,00020152	0,00020717	0,00192269	0,00147122	0,018067824	0,00021128	0,00022899	0,00060306	0,00035261	0,00190448	0	0	0,03316625	0
CS-M2 with Bag - Mean	0,01585455	0,04441818	0,00073636	0,00568182	0,000109091	0,01209091	0,02273636	0,00792727	0,06478182	0,00171818	0	0	0,0002	0
Standard Deviation	0,00026411	0,00092914	0,00031413	0,00072716	0,000183177	0,00025745	0,00053474	0,00033599	0,00353857	0,00027242	0	0	0,0004264	0
% change to Mean	0,00016658	0,00020918	0,00426596	0,0012798	0,016791201	0,00021293	0,00023519	0,00042385	0,00054623	0,00158554	#DIV/0!	#DIV/0!	0,02132007	0
Factor	1,22782875	1,42921954	1,65226337	1,0164	0,840277778	1,02901003	0,59266293	0,4478211	0,50116943	0,73236332	0	0	0,41666667	0
Standards Target value														
JMS-2 published values	0,0447	0,0166	0	0,0035	0,00E+00	6,50E-03	0,0454	0,0254	0,022	0,003	0	0	0	0,0003
JMS-2 - Mean	0,03706	0,01166	0,00092	0,00304	0	0,00582	0,03913	0,02428	0,02468	0	0,00024	0	0,00009	0
JMS-2 with Bag - Mean	0,03161429	0,0089	0,0006	0,00265714	0	0,0055	0,04618571	0,03357143	0,02631429	0	0,00091429	0	0,0001	0
Factor	1,17225486	1,31011236	1,53333333	1,14408602	0	1,05818182	0,84723167	0,72323404	0,93789359	0	0,2625	0	0,9	0
SARM 46 published values	0,0556	0,656	0	0,089	0,00E+00	0,0027	0,0025	0,0014	0,101	0	0	0,0055	0,0074	0
SARM 46 - Mean	0,04043	0,59825	0,00144	0,08307	0	0,00364	0,00386	0,00241	0,00516	0	0	0,00372	0,00248	0,00065
Standard Deviation	0,00163588	0,00694597	0,00122654	0,00564323	0	0,00031369	0,00026153	0,00039611	0,00038781	0	0	0,00040939	0,00045343	0,0013048
% change to Mean	0,00040462	0,0001161	0,00851764	0,00067933	0	0,00086178	0,00067755	0,00164359	0,00075158	0	0	0,00110051	0,00182835	0,02007383
SARM 46 with Bag - Mean	0,03186	0,42641	0	0,06768	0,00004	0,00288	0,00414	0,00337	0,0115	0	0,00015	0,00252	0,00215	0
Standard Deviation	0,00235253	0,02720494	0	0,00644559	0,00012	0,00039446	0,00039547	0,00052545	0,00095603	0	0,00045	0,00132272	0,00043417	0
% change to Mean	0,0007384	0,000638	0	0,00095236	0,03	0,00136966	0,00095525	0,00155921	0,00083133	0	0,03	0,00524891	0,00201938	0
Factor	1,26898933	1,40299243	0	1,22739362	0	1,26388889	0,93236715	0,71513353	0,44869565	0	0	1,47619048	1,15348837	0
Limit of Detection	0,003	0,001	0,001	0,001	0,001	0,001	0,001	0,001	0,001	0,002	0,003	0,004	0,003	0,007
Upper Limit	6,8	1,5	0,02	2,2	0,01	0,07	1,1	0,22	1,6	42,4	1,5	0,02	0,02	1,4

all values in mg/kg

sample	Sb	Ba	La	Ce	Hf	Ta	W	Pt	Au	Hg	Tl	Pb	Bi	Th
CS-M2 standard with plastic bag -1	0,0163	0,1617	0	0	0,0003	0	0	0	0	0	0	0,0666	0	0
CS-M2 standard with plastic bag -2	0,0207	0,1625	0	0,0113	0	0	0	0	0	0	0	0,0675	0	0,0014
CS-M2 standard with plastic bag -3	0,0152	0,1641	0,0095	0	0	0	0	0	0	0	0	0,0685	0	0
CS-M2 standard with plastic bag -4	0,0196	0,1622	0	0	0	0	0	0	0	0	0	0,0693	0,0016	0
CS-M2 standard with plastic bag -5	0,0192	0,1682	0	0,0121	0	0	0	0	0	0	0	0,0648	0	0,002
CS-M2 standard with plastic bag -6	0,0208	0,1669	0	0	0,0004	0	0	0	0	0	0	0,0661	0	0,0016
CS-M2 standard with plastic bag -7	0,0185	0,1574	0	0	0	0	0	0	0	0	0	0,0663	0	0,0021
CS-M2 standard with plastic bag -8	0,0165	0,1625	0,0093	0,0122	0	0	0	0	0	0	0	0,0685	0	0
CS-M2 standard with plastic bag -9	0,0156	0,162	0	0	0	0	0	0	0	0	0	0,0652	0	0
CS-M2 standard with plastic bag -10	0,0171	0,1547	0,0104	0	0	0	0	0	0	0	0	0,0649	0	0,0016
CS-M2 standard with plastic bag -11	0,0156	0,1572	0	0	0	0	0	0	0	0	0	0,0645	0	0,0016
CS-M2 standard -1	0,0158	0,0966	0	0	0,0003	0	0	0	0	0	0	0,0694	0	0,001
CS-M2 standard -2	0,0146	0,094	0	0	0	0	0	0	0	0	0	0,074	0	0,001
CS-M2 standard -3	0,0174	0,0947	0	0	0	0	0	0	0	0	0	0,0706	0	0,0012
CS-M2 standard -4	0,0135	0,0949	0	0	0	0	0	0	0	0	0	0,0715	0	0,0013
CS-M2 standard -5	0,0137	0,0961	0	0	0,0003	0	0	0	0	0	0	0,0718	0	0,0009
CS-M2 standard -6	0,0183	0,0962	0	0	0	0	0	0	0	0	0	0,0704	0	0
CS-M2 standard -7	0,0133	0,0939	0	0	0	0	0	0	0	0	0	0,0691	0	0
CS-M2 standard -8	0,0167	0,0942	0	0	0,0002	0	0,0034	0	0	0	0	0,0695	0,0012	0
CS-M2 standard -9	0,0144	0,0893	0	0	0	0	0,002	0	0	0	0	0,071	0	0,0016
CS-M2 standard -10	0,017	0,0866	0	0	0,0003	0	0	0	0	0	0	0,0695	0	0,0011
CS-M2 standard -11	0,0179	0,0937	0,0082	0,0138	0,0002	0	0,0028	0	0	0	0	0,0722	0	0
CS-M2 standard -12	0,0164	0,0973	0,0055	0	0,0003	0	0,0035	0	0	0	0	0,0733	0	0
JMS-2 standard -1	0	0,1577	0,0086	0,0164	0	0	0	0	0	0	0	0,0054	0,0013	0
JMS-2 standard -2	0,0027	0,1576	0,0095	0	0	0	0	0	0	0	0	0,0068	0	0
JMS-2 standard -3	0,0026	0,1574	0	0	0	0	0	0	0	0	0	0,0058	0	0
JMS-2 standard -4	0	0,1535	0	0	0	0	0	0	0	0	0	0,006	0	0
JMS-2 standard -5	0	0,1615	0,0115	0,0139	0	0	0,002	0	0	0	0	0,005	0	0,0017
JMS-2 standard -6	0	0,1555	0,0093	0,0105	0	0	0	0	0	0	0	0,0063	0	0,0009
JMS-2 standard -7	0	0,1589	0	0	0	0	0	0	0	0	0	0,0066	0	0
JMS-2 standard -8	0	0,1681	0	0,0143	0	0	0	0	0	0	0	0,0057	0	0
JMS-2 standard -9	0	0,1594	0,0111	0,0141	0	0	0	0	0	0	0	0,0056	0	0
JMS-2 standard -10	0	0,157	0	0	0	0	0	0	0	0	0	0,0071	0	0
JMS-2 standard with bag -1	0	0,2225	0	0,0114	0	0	0,0019	0	0	0	0	0,0061	0	0
JMS-2 standard with bag -2	0	0,2047	0,0135	0	0	0	0	0	0	0	0	0,006	0	0
JMS-2 standard with bag -3	0	0,1774	0,0155	0,0165	0	0	0	0	0	0	0	0,0048	0	0,0012
JMS-2 standard with bag -4	0	0,1936	0,0187	0,0146	0	0	0	0	0	0	0	0,0054	0	0,0009
JMS-2 standard with bag -5	0	0,2097	0,0196	0	0	0	0	0	0	0	0	0,0064	0	0
JMS-2 standard with bag -6	0	0,2095	0,0149	0,0113	0	0	0	0	0	0	0	0,0056	0	0
JMS-2 standard with bag -7	0	0,1942	0,0155	0	0	0	0	0	0	0	0	0,0058	0	0,0009
SARM 46 standard -1	0,0935	0,0189	0	0,0165	0,0022	0,0125	0,0085	0	0	0	0	1,2926	0	0
SARM 46 standard -2	0,095	0,0217	0	0,0203	0,0027	0,0117	0,0067	0	0	0	0	1,3062	0	0
SARM 46 standard -3	0,0951	0,0202	0	0	0,0027	0,0123	0	0	0	0,0015	0	1,3104	0,0039	0
SARM 46 standard -4	0,0945	0,0175	0	0	0,0032	0,0125	0	0	0	0,0015	0	1,2749	0,0056	0
SARM 46 standard -5	0,0952	0,0199	0	0,0144	0,0027	0,0131	0	0	0	0	0	1,297	0,0039	0
SARM 46 standard -6	0,0944	0,0207	0	0	0,0029	0,0106	0	0	0	0	0	1,2855	0	0
SARM 46 standard -7	0,0956	0,0195	0	0	0,0026	0,0096	0,0114	0	0	0	0	1,2868	0	0
SARM 46 standard -8	0,0923	0,0176	0	0	0,0029	0,0101	0,0075	0	0	0	0	1,301	0	0,0034
SARM 46 standard -9	0,0993	0,0162	0,0122	0,0155	0,002	0	0	0	0	0	0	1,2921	0	0

SM.5 Plastic Bag Experiment Results

sample	Sb	Ba	La	Ce	Hf	Ta	W	Pt	Au	Hg	Tl	Pb	Bi	Th
SARM 46 standard -10	0,095	0,0177	0	0	0,0022	0,0138	0,0067	0	0	0	0	1,3076	0	0
SARM 46 standard with bag -1	0,094	0,025	0	0	0,0021	0	0	0	0	0	0	1,1363	0	0
SARM 46 standard with bag -2	0,0897	0,0176	0	0,0178	0,0017	0,0114	0	0	0	0,0014	0	1,1167	0	0
SARM 46 standard with bag -3	0,0934	0,0206	0	0	0,0026	0	0	0	0	0	0	1,1142	0	0
SARM 46 standard with bag -4	0,0803	0,0131	0	0	0,0021	0	0,0133	0	0	0	0	0,8619	0	0
SARM 46 standard with bag -5	0,0868	0,02	0	0	0,0016	0	0,0146	0	0	0	0	1,1281	0,0049	0
SARM 46 standard with bag -6	0,0819	0,0215	0	0	0,003	0	0	0	0	0	0	1,0698	0	0
SARM 46 standard with bag -7	0,0767	0,0185	0	0	0,0019	0	0	0	0	0,0014	0	1,1129	0	0
SARM 46 standard with bag -8	0,0925	0,0227	0	0	0,002	0	0	0	0	0	0	1,1401	0,0033	0
SARM 46 standard with bag -9	0,0846	0,0166	0	0	0,0026	0	0	0	0	0	0	1,1109	0	0
SARM 46 standard with bag -10	0,0877	0,0203	0	0	0,002	0,0117	0	0	0	0	0	1,1294	0	0
CS-M2 Target value	NA	NA	NA	NA	NA	NA	NA	NA	NA	NA	NA	0,0711	NA	NA
CS-M2 - Mean	0,01575	0,09395833	0,00114167	0,00115	0,00013333	0	0,000975	0	0	0	0	0,071025	0,0001	0,000675
Standard Deviation	0,00171148	0,00296042	0,00261166	0,00381412	0,00013744	0	0,00142134	0	0	0	0	0,00152213	0,00033166	0,00059459
% change to Mean	0,00108665	0,00031508	0,02287585	0,03316625	0,01030776	0	0,01457785	0	0	0	0	0,00021431	0,03316625	0,0088088
CS-M2 with Bag - Mean	0,01773636	0,16176364	0,00265455	0,00323636	6,3636E-05	0	0	0	0	0	0	0,06656364	0,00014545	0,00093636
Standard Deviation	0,00199831	0,00386553	0,00434205	0,00528914	0,00013667	0	0	0	0	0	0	0,00159959	0,00045997	0,00087415
% change to Mean	0,00112667	0,00023896	0,01635703	0,01634286	0,02147614	0	0	0	0	0	0	0,00024031	0,03162278	0,00933555
Factor	0,88800615	0,58083717	0,43007991	0,35533708	2,0952381	0	0	0	0	0	0	1,06702404	0,6875	0,72087379
Standards Target value														
JMS-2 published values	0,0005	0,1856	0,01325	0,0156	0,0005	0,0002	0	0	0	0	0	0,0088	0	0,00137
JMS-2 - Mean	0,00053	0,15866	0,005	0,00692	0	0	0,0002	0	0	0	0	0,00603	0,00013	0,00026
JMS-2 with Bag - Mean	0	0,20165714	0,01395714	0,00768571	0	0	0,00027143	0	0	0	0	0,00572857	0	0,00042857
Factor	0	0,78678096	0,35823951	0,90037175	0	0	0,73684211	0	0	0	0	1,05261845	0	0,60666667
SARM 46 published values	0,118	0,0172	0,0019	0,0066	0,0003	0,00003	<0.0020	0	0,00001	0,0007	0	1,3	0	0,001
SARM 46 - Mean	0,09499	0,01899	0,00122	0,00667	0,00261	0,01062	0,00408	0	0	0,0003	0	1,29541	0,00134	0,00034
Standard Deviation	0,00170438	0,0016257	0,00366	0,00828891	0,00035341	0,00376053	0,00426352	0	0	0,0006	0	0,01065528	0,00209342	0,00102
% change to Mean	0,00017943	0,00085608	0,03	0,01242716	0,00135407	0,00354099	0,0104498	0	0	0,02	0	8,2254E-05	0,01562252	0,03
SARM 46 with Bag - Mean	0,08676	0,01959	0	0,00178	0,00216	0,00231	0,00279	0	0	0,00028	0	1,09203	0,00082	0
Standard Deviation	0,0055696	0,00317	0	0,00534	0,00041761	0,00462049	0,00558757	0	0	0,00056	0	0,07893913	0,00167857	0
% change to Mean	0,00064195	0,00161817	0	0,03	0,00193339	0,02000211	0,02002712	0	0	0,02	0	0,00072287	0,02047038	0
Factor	1,09485938	0,96937213	0	3,74719101	1,20833333	4,5974026	1,46236559	0	0	1,07142857	0	1,1862403	1,63414634	0
Limit of Detection	0,01	0,015	0,013	0,02	0,001	0,014	0,004	0,005	0,003	0,003	0,002	0,002	0,002	0,002
Upper Limit	3,6	2,7	3	4,1	1,1	0,22	1,1	0,18	0,1	0,07	0,02	1,4	0,3	0,7

all values in mg/kg

sample	U
CS-M2 standard with plastic bag -1	0
CS-M2 standard with plastic bag -2	0,0027
CS-M2 standard with plastic bag -3	0
CS-M2 standard with plastic bag -4	0
CS-M2 standard with plastic bag -5	0,0047
CS-M2 standard with plastic bag -6	0,0028
CS-M2 standard with plastic bag -7	0
CS-M2 standard with plastic bag -8	0,0032
CS-M2 standard with plastic bag -9	0
CS-M2 standard with plastic bag -10	0
CS-M2 standard with plastic bag -11	0,003
CS-M2 standard -1	0
CS-M2 standard -2	0,0007
CS-M2 standard -3	0,0009
CS-M2 standard -4	0,0011
CS-M2 standard -5	0,0007
CS-M2 standard -6	0
CS-M2 standard -7	0,0006
CS-M2 standard -8	0
CS-M2 standard -9	0,0014
CS-M2 standard -10	0,0009
CS-M2 standard -11	0
CS-M2 standard -12	0,0009
JMS-2 standard -1	0
JMS-2 standard -2	0
JMS-2 standard -3	0
JMS-2 standard -4	0
JMS-2 standard -5	0
JMS-2 standard -6	0
JMS-2 standard -7	0
JMS-2 standard -8	0
JMS-2 standard -9	0
JMS-2 standard -10	0
JMS-2 standard with bag -1	0
JMS-2 standard with bag -2	0
JMS-2 standard with bag -3	0
JMS-2 standard with bag -4	0
JMS-2 standard with bag -5	0
JMS-2 standard with bag -6	0
JMS-2 standard with bag -7	0
SARM 46 standard -1	0
SARM 46 standard -2	0
SARM 46 standard -3	0
SARM 46 standard -4	0
SARM 46 standard -5	0
SARM 46 standard -6	0
SARM 46 standard -7	0
SARM 46 standard -8	0
SARM 46 standard -9	0

	Ca	Cr	Ni	Cu	Zn	As	Cd	Hg	Pb
CS-M2 Target value	0,55	NA	NA	0,02	NA	NA	NA	NA	0,0711
CS-M2	0,533475	0,00573333	0,0047	0,01946667	0,06348333	0,005775	8,3333E-05	0	0,071025
CS-M2 with Bag	0,16821818	0,00055455	0,00475455	0,01585455	0,04441818	0,00568182	0,0002	0	0,06656364
Difference	0,36525682	0,00517879	5,4545E-05	0,00361212	0,01906515	9,3182E-05	0,00011667	0	0,00446136
Change in %	-68,4674667	-90,3276956	1,16054159	-18,5554172	-30,031743	-1,61353798	140	0	-6,281399
Correction factor for plastic bag	3,17132782	10,3387978	0,98852772	1,22782875	1,42921954	1,0164	0,41666667	0	1,06702404
JMS-2 published values	4,68	0,0078	0,0311	0,0447	0,0166	0,0035	0	0	0,0088
JMS-2 - Mean	2,56958	0	0,03034	0,03706	0,01166	0,00304	0,00009	0	0,00603
JMS-2 with Bag - Mean	1,78282857	0	0,02892857	0,03161429	0,0089	0,00265714	0,0001	0	0,00572857
Difference	0,78675143	0	0,00141143	0,00544571	0,00276	0,00038286	0,00001	0	0,00030143
Change in %	-30,6178998	0	-4,6520388	-14,6943181	-23,670669	-12,593985	11,1111111	0	-4,99881545
Correction factor for plastic bag	1,44129393	0	1,04879012	1,17225486	1,31011236	1,14408602	0,9	0	1,05261845
SARM 46 published value	0,943404	0,0559	0,0127	0,0556	0,656	0,089	0,0074	0,0007	1,3
SARM 46	1,044	0,06034	0,02616	0,04043	0,59825	0,08307	0,00248	0,0003	1,29541
SARM 46 with bag	0,72121	0,05156	0,0231	0,03186	0,42641	0,06768	0,00215	0,00028	1,09203
Difference	0,32279	0,00878	0,00306	0,00857	0,17184	0,01539	0,00033	0,00002	0,20338
Change in %	-30,9185824	-14,5508784	-11,6972477	-21,1971308	-28,7237777	-13,3064516	-13,3064516	-6,66666667	-15,7000486
Correction factor for plastic bag	1,44756728	1,17028704	1,13246753	1,26898933	1,40299243	1,22739362	1,15348837	1,07142857	1,1862403
**Proposed correction factor**	**1,44**	**1,17**	**1,05**	**1,2**	**1,4**	**1,2**	**1,15**	**No factor**	**1,18**

SM.5 Plastic Bag Experiment Results

sample	U
SARM 46 standard -10	0
SARM 46 standard with bag -1	0
SARM 46 standard with bag -2	0
SARM 46 standard with bag -3	0
SARM 46 standard with bag -4	0
SARM 46 standard with bag -5	0
SARM 46 standard with bag -6	0
SARM 46 standard with bag -7	0
SARM 46 standard with bag -8	0
SARM 46 standard with bag -9	0
SARM 46 standard with bag -10	0

CS-M2 Target value	NA
CS-M2 - Mean	0,0006
Standard Deviation	0,00046726
% change to Mean	0,00778769
CS-M2 with Bag - Mean	0,00149091
Standard Deviation	0,00170585
% change to Mean	0,01144166
Factor	0,40243902

Standards Target value	
JMS-2 published values	0,0003
JMS-2 - Mean	0
JMS-2 with Bag - Mean	0
Factor	0

SARM 46 published values	0,0001
SARM 46 - Mean	0
Standard Deviation	0
% change to Mean	0
SARM 46 with Bag - Mean	0
Standard Deviation	0
% change to Mean	0
Factor	0

Limit of Detection	0,001
Upper Limit	0,2

all values in mg/kg
