# Supplementary material for: Portable X-ray fluorescence (pXRF) analysis of heavy metals in contaminated soils: a case study from Varta Insel, Hagen
Source: Environ Geochem Health. 2026 Oct 27;48(16):667. doi: 10.1007/s10653-026-03538-5 (PMC13714078; doi:10.1007/s10653-026-03538-5)
Supplement: Supplementary file 2 [file 10653_2026_3538_MOESM1_ESM.docx]

**SUPPLEMENTARY INFORMATION**

**SM 1: RStudio Script**

library(viridis)

library(ggplot2)	# plotting library

library(tidyverse) # reshape2 package to transform data frames for ggplot

mycol = viridis(11, alpha = 0.75, option = "H") # generate a color palette

keV = seq(0,50, length.out = 2048) # keV sequence of pXRF

\# Measurements 3904 through 3914 were done through bags

bags = c(3904:3914)

bags = as.character(bags)

filenames = paste("Spectrum_0", bags,"-GeoExploration_041223_120356.txt", sep = "")

bags_data = matrix(nrow = 2048, ncol = 11)

\# the spectra are read and stored in matrix bags_data

for (i in 1:11){

bags_data[,i] = as.matrix(read.delim(filenames[i], header = F))

}

\# plot all 11 spectra on top of each other, to check that they all look alike

plot(keV, bags_data[,1], col = "blue", type = "l", ylab = "Measurement 1")

dev.off()

plot(keV, bags_data[,1], col = mycol[1], type = "l", ylab = "Counts - With plastc Bag", xlim = c(0, 12))

for (i in 2:11){

lines(keV, bags_data[,i], col = mycol[i], lty = i, lwd = 1+(0.1*i))

}

plot(keV, bags_data[,1], col = mycol[1], type = "l", ylab = "Counts - With plastc Bag", xlim = c(0, 12))

for (i in 2:11){

lines(keV, bags_data[,i], col = mycol[i], lty = i, lwd = 1+(0.1*i))

}

\# ggplot, save types for the later legend

CType = c("#01" = "yellow", "#02" = "orange", "#03" = "green", "#04" = "red", "#05" = "cyan", "#06" = "blue")

LType = c("#01" = "solid", "#02" = "longdash", "#03" = "twodash", "#04" = "dotdash", "#05" = "dashed", "#06" = "dotted")

\# plot the 1st 6 measurements on ggplot with different line types and colors

testdata <- data.frame(keV, bags_data)

ggplot(testdata) +

xlim(0, 13)+

geom_line(aes(x = keV, y = X1, color = "#01", linetype = "#01")) +

geom_line(aes(x = keV, y = X2, color = "#02", linetype = "#02")) +

geom_line(aes(x = keV, y = X3, color = "#03", linetype = "#03")) +

geom_line(aes(x = keV, y = X4, color = "#04", linetype = "#04")) +

geom_line(aes(x = keV, y = X5, color = "#05", linetype = "#05")) +

geom_line(aes(x = keV, y = X6, color = "#06", linetype = "#06")) +

scale_color_manual(name = "Plastic Bag Experiment", values = CType)+

scale_linetype_manual(name = "Plastic Bag Experiment", values = LType)+

\# ggtitle("Plastic Bag Experiment") +

ylab("Counts") +

xlab("Energy [keV]") +

theme_bw() +

theme(text = element_text(size = 14))+

theme(legend.position = c(.8, .7), legend.key.width = unit(2, "cm"))

\# save plot

ggsave(filename = "pXRF PlasticBagExperiment 6 combined NEW.png", dpi = 600, width = NA, height = NA)

\# rm(testdata)

\# calculate the average

bags_data = rowMeans(bags_data)

plot(keV, bags_data, col = mycol[1], type = "l")

\# same for the no-bag measurements.

nobags = c(3915:3924)

nobags = as.character(nobags)

filenames = paste("Spectrum_0", nobags,"-GeoExploration_041223_120356.txt", sep = "")

nobags_data = matrix(nrow = 2048, ncol = 10)

for (i in 1:10){

nobags_data[,i] = as.matrix(read.delim(filenames[i], header = F))

}

plot(nobags_data[,1], col = mycol[1], type = "l")

for (i in 2:10){

lines(nobags_data[,i], col = mycol[i])

}

\# ggplot of the 1st 6 no bag measurements

\# plot the 1st 6 measurements on ggplot with different linetypes and colors

testdata <- data.frame(keV, nobags_data) # dataframe with new data for ggplot

CType = c("#01" = "magenta", "#02" = "chartreuse3", "#03" = "gold3", "#04" = "firebrick3", "#05" = "royalblue4", "#06" = "aquamarine")

ggplot(testdata) +

xlim(0, 13)+

geom_line(aes(x = keV, y = X1, color = "#01", linetype = "#01")) +

geom_line(aes(x = keV, y = X2, color = "#02", linetype = "#02")) +

geom_line(aes(x = keV, y = X3, color = "#03", linetype = "#03")) +

geom_line(aes(x = keV, y = X4, color = "#04", linetype = "#04")) +

geom_line(aes(x = keV, y = X5, color = "#05", linetype = "#05")) +

geom_line(aes(x = keV, y = X6, color = "#06", linetype = "#06")) +

scale_color_manual(name = "Standard Measurment", values = CType)+

scale_linetype_manual(name = "Standard Measurment", values = LType)+

\# ggtitle("Standard Measurment") +

ylab("Counts") +

xlab("Energy [keV]") +

theme_bw() +

theme(text = element_text(size = 14))+

theme(legend.position = c(.8, .7), legend.key.width = unit(2, "cm"))

\# save plot

ggsave(filename = "pXRF NO BAG 6 combined NEW.png", dpi = 600, width = NA, height = NA)

rm(testdata)

\# averages of no bag data

nobags_data = rowMeans(nobags_data)

plot(keV, nobags_data, col = mycol[3], type = "l")

\# compare bags vs. nobags

plot(keV, nobags_data, col = "red", type = "l", xlim = c(0.5, 13), ylab = "Count Averages")

lines(keV, bags_data, col = mycol[3])

\# ggplot variant

CType = c("No Bag" = "red", "Plastic Bag" = "navy")

LType = c("No Bag" = "solid", "Plastic Bag" = "longdash")

ggplot(data.frame(keV, nobags_data, bags_data)) +

geom_line(aes(x = keV, y = nobags_data, color = "No Bag", linetype = "No Bag"))+

geom_line(aes(x = keV, y = bags_data, color = "Plastic Bag", linetype = "Plastic Bag"))+

xlim(0, 18) +

scale_color_manual(name = "Mean of Measurements", values = CType)+

scale_linetype_manual(name = "Mean of Measurements", values = LType)+

ylab("Counts") +

xlab("Energy [keV]") +

theme_bw() +

theme(legend.position = c(.8, .7), legend.key.width = unit(2, "cm"), text = element_text(size = 14))

\# save plot version 2.0

ggsave(filename = "Difference Means NoBag-Bag NEW.png", dpi = 600, width = NA, height = NA)

\#### pXRF x ICP-OES plots v1.7 ####

library(readxl)	# read excel files

library(dplyr)

Data_combined <- read_xlsx("pXRF x Lab combined.xlsx")

\# Create an empty list to store the plots

for_data <- data.frame()

plots_list <- list()

CType = c("Consensus Over Limit" = "red3", "Consensus Under Limit" = "green4", "Controversial" = "orange3")

\# for loop to create plots using ggplot2

for (column_name in c("Cr", "Ni", "Zn", "Pb", "Cd","Cu","Hg","As")) {

\# Subset data for the current column and type pXRF

pXRF_data <- Data_combined[1:106, column_name]

\# Subset data for the current column and type Lab

Lab_data <- Data_combined[107:212, column_name]

for_data <- cbind(pXRF_data, Lab_data, Con = Data_combined[1:106, "Con"])

colnames(for_data) <- c("pXRF","Lab","Confidence")

\# Define parameters for geom_rect based on column_name

if (column_name == "Cr") {

xmaxTemp <- 200

ymaxTemp <- 200

LOD <- 140

} else if (column_name == "Ni") {

xmaxTemp <- 900

ymaxTemp <- 900

LOD <- 20

} else if (column_name == "Pb") {

xmaxTemp <- 2000

ymaxTemp <- 2000

LOD <- 20

} else if (column_name == "Cd") {

xmaxTemp <- 60

ymaxTemp <- 60

LOD <- 30

} else if (column_name == "Hg") {

xmaxTemp <- 100

ymaxTemp <- 100

LOD <- 30

} else if (column_name == "As") {

xmaxTemp <- 140

ymaxTemp <- 140

LOD <- 10

} else if (column_name == "Zn") {

xmaxTemp <- -1

ymaxTemp <- -1

LOD <- 10

} else {

xmaxTemp <- -1

ymaxTemp <- -1

LOD <- 30}

\# Create the plot

plot <- ggplot(for_data) +

geom_abline(intercept = 0, slope = 1, color = "blue")+ # Add y = x + 0 line

geom_vline(xintercept = xmaxTemp, color = "black", linetype = "dashed") +

geom_hline(yintercept = ymaxTemp, color = "black", linetype = "dashed") +

geom_point(aes(pXRF, Lab, color=ifelse(pXRF>xmaxTemp & Lab>ymaxTemp,"Consensus Over Limit", ifelse(pXRF<xmaxTemp & Lab<ymaxTemp,"Consensus Under Limit","Controversial"))))+

scale_color_manual(name = "pXRF x ICP-OES", values = CType)+

ylab(paste("Lab -", column_name,"[mg/kg]")) +

xlab(paste("pXRF -", column_name,"[mg/kg]")) +

geom_segment(x = LOD, y = -100, xend = LOD, yend = LOD, color = "navy", linetype = "dotdash") + # Add blue vertical line for LOD

theme_bw()+

theme(text = element_text(size = 14))

plots_list[[column_name]] <- plot	# Add the plot to the list

print(plot) # display the plots

ggsave(file = (paste("pXRF x ICPOES", column_name,".png")), dpi = 600)}# save the plots

rm(for_data, xmaxTemp, ymaxTemp, LOD) # remove for_data, xmaxTemp, ymaxTemp

**SM 2: Python Script**

import pandas as pd

import matplotlib.pyplot as plt

from itertools import chain

\# File paths

onsite_data_file = './data/Zweier_Vergleich_onSite.xlsx'

lab_data_file = './data/Zweier_Vergleich_LabpXRF.xlsx'

icpoes_data_file = './data/Dreier_Vergleich_ICPOES.xlsx'

\# Elements to plot

elements = ['Cr', 'Ni', 'Cu', 'Zn', 'As', 'Cd', 'Sb', 'Hg', 'Pb']

def main():

\# Load data from Excel files

onsite_data = pd.read_excel(onsite_data_file)

lab_data = pd.read_excel(lab_data_file)

icpoes_data = pd.read_excel(icpoes_data_file)

\# Group data by 'Point' and store in dictionaries

groups = onsite_data.groupby('Point')

onsite_by_datapoints = {datapoint: group for datapoint, group in groups}

groups = lab_data.groupby('Point')

lab_data_by_datapoints = {datapoint: group for datapoint, group in groups}

groups = icpoes_data.groupby('Point')

icpoes_data_by_datapoints = {datapoint: group for datapoint, group in groups}

\# Find the maximum value for each element to scale the y-axes

max_value_by_elements = {}

for element in elements:

current_max_value = max(chain(onsite_data[element], lab_data[element], icpoes_data[element]))

max_value_by_elements[element] = current_max_value

\# Create subplots for each element

for i, element in enumerate(elements):

fig, axes = plt.subplots(nrows=2, ncols=4, figsize=(20, 10)) # Adjust to 2 rows and 4 columns

\# Iterate over data points and create subplots

for j, current_datapoint in enumerate(list(onsite_by_datapoints.keys())):

selected_columns = ['Depth', 'Point', 'Lower', 'Upper' , element]

lab_data_datapoint = lab_data_by_datapoints[current_datapoint][selected_columns]

onsite_data_datapoint = onsite_by_datapoints[current_datapoint][selected_columns]

icpoes_data_by_datapoint = icpoes_data_by_datapoints[current_datapoint][selected_columns]

ax = axes[j // 4, j % 4] # Update to 4 columns

\# Set the font size of the x-axis and y-axis tick labels

ax.tick_params(axis='x', labelsize=16)

ax.tick_params(axis='y', labelsize=16)

\# Plot onsite data

line1, = ax.plot(onsite_data_datapoint['Depth'], onsite_data_datapoint[element], color='navy', label='pXRF on-Site data' if j == 0 else "")

\# Plot lab data

for _, row in lab_data_datapoint.iterrows():

line2 = ax.plot([row['Lower'], row['Upper']], [row[element]] * 2, color='darkorange', linestyle='-.', label='pXRF Lab data' if j == 0 else "")

for _, row in icpoes_data_by_datapoint.iterrows():

line3 = ax.plot([row['Lower'], row['Upper']], [row[element]] * 2, color='forestgreen', linestyle='--', label='ICP-OES data' if j == 0 else "")

ax.set_title(f'#{current_datapoint}', loc='left', fontweight='bold', fontsize=20)

ax.set_ylabel(f'{element} concentration [mg/kg]', fontsize = 15)

ax.set_xlabel('Depth [m]', fontsize = 15)

ax.set_ylim(-10, max_value_by_elements[element]*1.06) # Y-axis limit

if element == 'Cd' : (ax.set_ylim(-1 , max_value_by_elements[element]*1.06)) # Cd is too close to 0 for -10 ylim

if element == 'Pb' : (ax.set_ylim(-120 , max_value_by_elements[element]*1.06)) # Pb is too large for -10 ylim

\# Legend in the last subplot

ax = axes[-2, -1]

handles = [

plt.Line2D([], [], color='navy', label='On-Site (pXRF)'),

plt.Line2D([], [], color='darkorange', linestyle='-.', label='Laboratory (pXRF)'),

plt.Line2D([], [], color='forestgreen', linestyle='--', label='Laboratory (ICP-OES)')

]

ax.legend(handles=handles, loc='best', frameon=False, fontsize=18)

\# Add element abbreviation in 1st plot

ax = axes[-2, -4]

ax.text(s = f'{element}', x=1.95, y=max_value_by_elements[element]*0.86, ha='left', fontweight='bold', fontsize=38)

\# Layout anpassen

plt.tight_layout()

\# Save as SVG or PNG

plt.savefig(f'./output/new_4x2_element_{element}.svg', format='svg') # png/svg with dpi = 600

if __name__ == '__main__':

main()

**SM 3: Analytical range of the Bruker Titan S1 in GeoExploration mode**

| [mg/kg] | **MgO** | **Al2O3** | **SiO2** | **P** | **S** | **Cl** | **K2O** | **Ca** | **Ti** | **V** | **Cr** |
| --- | --- | --- | --- | --- | --- | --- | --- | --- | --- | --- | --- |
| Lower limit | 5000 | 1200 | 1000 | 150 | 300 | 250 | 160 | 100 | 100 | 80 | 140 |
| Upper limit | 700,000 | 690,000 | 1000,000 | 132,000 | 37,000 | 40,000 | 154,000 | 311,000 | 194,000 | 107,000 | 24,000 |
|  | **Mn** | **Fe** | **Ni** | **Cu** | **Zn** | **Ga** | **As** | **Se** | **Rb** | **Sr** | **Y** |
| Lower limit | 50 | 50 | 20 | 30 | 10 | 10 | 10 | 10 | 10 | 10 | 10 |
| Upper limit | 160,000 | 345,000 | 14,000 | 68,000 | 15,000 | 200 | 22,000 | 100 | 700 | 11,000 | 2,200 |
|  | **Zr** | **Nb** | **Mo** | **Ag** | **Cd** | **Sn** | **Sb** | **Ba** | **La** | **Ce** | **Hf** |
| Lower limit | 10 | 20 | 30 | 40 | 30 | 70 | 100 | 150 | 130 | 200 | 10 |
| Upper limit | 16,000 | 424,000 | 15,000 | 200 | 200 | 14,000 | 36,000 | 27,000 | 30,000 | 41,000 | 11,000 |
|  | **Ta** | **W** | **Pt** | **Au** | **Hg** | **Tl** | **Pb** | **Bi** | **Th** | **U** |  |
| Lower limit | 140 | 40 | 50 | 30 | 30 | 20 | 20 | 20 | 20 | 10 |  |
| Upper limit | 2,200 | 11,000 | 1,800 | 1,000 | 700 | 200 | 14,000 | 3,000 | 7,000 | 2,000 |  |

The SM.3 table shows the concentration range with upper and lower limits for which the GeoExploration mode is calibrated as specified by Bruiker. The calibration is intended for elemental analysis of geological samples. For best results sample should be firmly packed in a sample cup using 4 µm Prolene film. (Bruker S1 Titan 800 – 2020, User Manual)

**SM 4: Antimony (Sb) concentration (mg/kg) in relation to depth (m), on site - Lab**

*(8 pages)*

*(18 pages)*

*(18 pages)*

*(18 pages)*

**SM 5: Plastic Bag Experiment Results (separate file)**
